# Unraveling reticulate evolution in North American *Dryopteris* (Dryopteridaceae)

**DOI:** 10.1186/1471-2148-12-104

**Published:** 2012-06-30

**Authors:** Emily B Sessa, Elizabeth A Zimmer, Thomas J Givnish

**Affiliations:** 1 Department of Botany, University of Wisconsin-Madison, 430 Lincoln Drive, Madison, WI, 53706, USA; 2 Department of Botany, National Museum of Natural History, MRC 166, Smithsonian Institution, Washington, DC, 20013-7012, USA

**Keywords:** Ferns, Divergence time estimates, Genetic distances, Hybridization, Introgression, Phylogeny, Polyploidy

## Abstract

**Background:**

The thirteen species of *Dryopteris* in North America have long been suspected of having undergone a complicated history of reticulate evolution via allopolyploid hybridization. Various explanations for the origins of the allopolyploid taxa have been suggested, and though most lines of evidence have supported the so-called “semicristata” hypothesis, contention over the group’s history has continued in several recent, conflicting studies.

**Results:**

Sequence data from nine plastid and two nuclear markers were collected from 73 accessions representing 35 species of *Dryopteris.* Sequences from each of the allopolyploids are most closely related to their progenitor species as predicted by the “semicristata” hypothesis. Allotetraploid *D. campyloptera* appears to be derived from a hybrid between diploid *D. expansa* and *D. intermedia*; *D. celsa,* from diploid *D. ludoviciana* x *D. goldiana*; and *D. carthusiana* and *D. cristata,* from diploid *“D. semicristata”* x *D. intermedia* and *D. ludoviciana,* respectively. Allohexaploid *D. clintoniana* appears to be derived from *D. cristata* x *D.goldiana.* The earliest estimated dates of formation of the allopolyploids, based on divergence time analyses, were within the last 6 Ma. We found no evidence for recurrent formation of any of the allopolyploids. The sexual allopolyploid taxa are derived from crosses between parents that show intermediate levels of genetic divergence relative to all pairs of potential progenitors. In addition, the four allotetraploids are transgressive with respect to geographic range relative to one or both of their parents (their ranges extend beyond those of the parents), suggesting that ecological advantages in novel habitats or regions may promote long-term regional coexistence of the hybrid taxa with their progenitors.

**Conclusions:**

This study provides the first thorough evaluation of the North American complex of woodferns using extensive sampling of taxa and genetic markers. Phylogenies produced from each of three datasets (one plastid and two nuclear) support the “semicristata” hypothesis, including the existence of a missing diploid progenitor, and allow us to reject all competing hypotheses. This study demonstrates the value of using multiple, biparentally inherited markers to evaluate reticulate complexes, assess the frequency of recurrent polyploidization, and determine the relative importance of introgression vs. hybridization in shaping the histories of such groups.

## Background

Hybridization and allopolyploidy are widely recognized as dominant forces shaping the evolutionary histories of many organisms, especially plants [1-3]. These phenomena may distort the patterns of dichotomous branching typically recovered by phylogenetic analyses, and lead to non-bifurcating, or reticulate, evolutionary histories that can be difficult to untangle and interpret [4]. Polyploidization is particularly rampant among ferns, which have fewer barriers to interspecific hybridization than angiosperms [5-7]. As many as 33% of extant leptosporangiate fern species are thought to be the products of recent polyploidization [2]. Reticulate complexes comprising multiple species at various ploidy levels have been identified in many genera over the years, including *Asplenium*[8], *Equisetum*[9], and *Astrolepis*[10], but the most intriguing case of reticulate evolution may be presented by the North American woodfern complex (*Dryopteris*, Dryopteridaceae).

*Dryopteris* is a large genus (ca. 225 species) with a nearly cosmopolitan distribution [11], including thirteen species in North America north of Mexico [12]. This latter assemblage is one of the most widely studied groups of ferns in North America, and has long been thought to involve extensive reticulate evolution via allopolyploid hybridization [12-14]. The group includes seven sexual diploid taxa, five sexual tetraploids, one sexual hexaploid, and 29 sterile hybrids (more than are known from any other fern genus in North America [15]). Recently, Sessa et al. [16,17] demonstrated that the North American (NA) sexual taxa are not monophyletic, and that almost all of the diploids are more closely related to Asian, African, or European taxa, from which they have diverged over the last 10 million years (Ma). Among the sexual species, nine have been hypothesized to be part of a “reticulate complex” that has generated much interest in *Dryopteris* among botanists over the last century [14,18]. The complex consists of four allotetraploids (*D. campyloptera, D. celsa. D. carthusiana,* and *D. cristata,* the latter two also native to Europe)*,* the allohexaploid *D. clintoniana*, and four putative diploid parents (*D. expansa, D. intermedia, D. ludoviciana,* and *D. goldiana).* A fifth North American allotetraploid, *D. filix-mas* (also native to Europe), is not part of the reticulate complex, though its origins have also proven perplexing [19].

**Figure 1:**
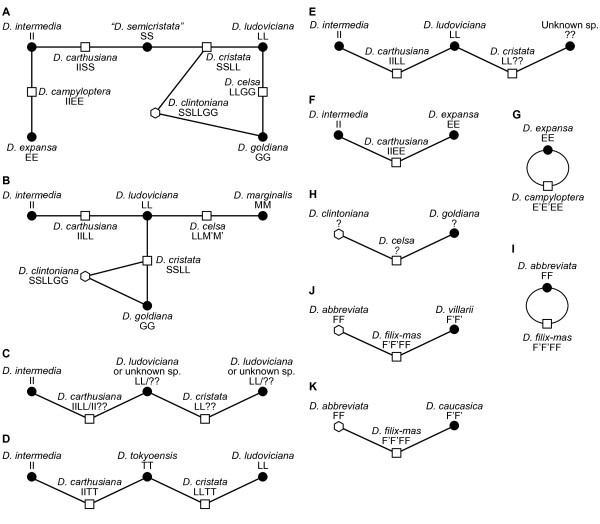
**Various hypotheses of the parentage of the *Dryopteris *polyploids in North America.A**) The “semicristata” hypothesis [12,14]. **B**) The “reinterpretation” hypothesis [37]. **CF**) Alternate hypotheses for the putative offspring of “*D. semicristata*” that employ only extant taxa [29,40,43,49]. **G**) Alternate hypothesis for *D. campyloptera*[27]. **H**) Alternate hypothesis for *D. celsa*[20]. **IK**) Hypotheses for *D. filix-mas*[30,33,50]. G and I indicate proposed instances of autopolyploidy. Diploids are depicted as solid circles, tetraploids as open squares, and hexaploids as open hexagons. Lines connect polyploids with their putative parental taxa. Inferred genomes are indicated as letters below taxon names. For ease of comparison, all diagrams use the same labeling scheme (e.g. II = *D. intermedia*, etc.), although several of the original publications employed an A, B, C, etc. labeling scheme for the various genomes. See Table 1 for additional references to hypotheses.

The parentage of the polyploids in the NA reticulate complex became the subject of intense study and debate beginning early in the 20th century, and various lines of evidence over the years have led to the development of several hypotheses to account for the origins of the allopolyploids (Table 1, Figure 1). Most evidence to date, including morphological and cytological observations, chemotaxonomy, spore morphology, chromatographic analyses, isozyme analyses, plastid restriction site analyses, and phylogenetic analysis of plastid and nuclear DNA sequences, has converged on support for the so-called “semicristata” hypothesis (Figure 1A). This scenario includes the four diploids previously mentioned as parents of the four allotetraploids, but invokes an additional diploid – *“D. semicristata”* – as a putatively extinct progenitor of two of the latter. The allohexaploid *D. clintoniana* is hypothesized to have formed by hybridization between the allotetraploid *D. cristata* and the diploid *D. goldiana.*

**Table 1:** **Summary of previous studies on North American *Dryopteris***

**Year**	**Reference**	**Hypothesis in Figure**1**that is supported, in whole or in part**	**Type of Observation/Study**
1953	Crane [20]	H	Spore morphology
1953	Manton & Walker [21]	I, J, or K for *D. filix-mas*; A for others	Cytological observations
1955	Walker [13]	A	Cytological observations
1959	Walker [22]	A	Cytological observations
1961	Walker [23]	A	Cytological observations
1962	Walker [24]	A	Cytological observations
1962	Wagner & Hagenah [25]	A	Morphology
1963	Wagner [26]	A	Morphology, cytological observations
1969	Wagner, Wagner, Hagenah [18]	A	Morphology, cytological observations
1969	Widén & Britton [27]	F for *D. campyloptera*; A for others	Cytological observations, chromatography, morphology
1969	Walker [28]	A	Cytological observations
1969	Widén & Sorsa [29]	G	Cytological observations, chromatography
1971	Widén et al. [30]	J	Chromatography
1971	Widén and Britton [31]	F for *D. campyloptera*; A for others	Cytological observations, chromatography
1971	Widén and Britton [32]	A	Cytological observations, chromatography
1971	Widén and Britton [33]	I	Cytological observations, chromatography
1972	Britton [34]	A	Spore morphology
1972	Fraser-Jenkins & Corley [35]	K	Morphology
1974	Britton & Widén [36]	A	Cytological observations, chromatography
1975	Hickok & Klekowski [37]	B	Cytological observations
1976	Fraser-Jenkins [38]	K	Cytological observations
1977	Gibby [39]	A	Cytological observations
1977	Gibby & Walker [40]	C	Cytological observations
1978	Gibby, Widén, Widén [41]	C	Cytological observations, chromatography
1983	Petersen & Fairbrothers [42]	A	Chromatography
1985	Widén & Britton [43]	D	Chromatography
1985	Werth [8]	A	Allozyme
1986	Viane [44]	A	Trichome morphology
1989	Werth [45]	A	Isozyme analyses
1989	Werth & Kuhn [45]	A	Morphology
1991	Werth [46]	A	Isozyme analyses
1992	Hutton [47]	A	plastid restriction site analyses
2010	Stein et al. [48]	A	Isozyme analyses, plastid restriction site analyses
2011	Juslen et al. [49]	A (plastid), E (nuclear)	plastid, nuclear sequence data
2012	Sessa et al. [16]	A	plastid sequence data
2012	Sessa et al. [17]	A	plastid, nuclear sequence data

For each publication, the type of observation or study is given, along with the hypothesis that was supported by the data, as presented in Figure 1.

A “missing diploid” is an inconvenient entity for systematists, and so *“D. semicristata”* has been a source of some skepticism since its existence was first postulated by Stanley Walker in the late 1950s [13,22]. The base chromosome number in *Dryopteris* is n = 41 [51] and Walker, in his cytological observations of the hybrid *D.* x *uliginosa,* a cross between the tetraploids *D. carthusiana* and *D. cristata,* noted that the hybrid showed 41 bivalents and 82 univalents at metaphase, indicating that the two tetraploids shared one genome in common, and each also contained a second, unrelated genome (donated by *D. intermedia* and *D. ludoviciana*, respectively, in Walker’s “semicristata” scheme). Cytological studies of additional species and hybrids were unable to attribute the shared genome to an extant taxon, and so the “missing diploid” was postulated to account for it [23,27]. The existence of the missing taxon has been supported by nearly all subsequent lines of evidence, but attempts have long been made to reinterpret the available data in support of hypotheses that involve only extant parental taxa (Figure 1B–E). Recently Juslen et al. [49] claimed, based on sequences of the nuclear marker *pgiC*, that *D. ludoviciana* is in fact the missing common ancestor of *D. carthusiana* and *D. cristata* (Figure 1E). In contrast, Sessa et al. [17] produced a phylogeny, also based on *pgiC* sequences, that supported the “semicristata” hypothesis. Plastid sequence data have so far provided support for one of each of the polyploids’ predicted parents in the “semicristata” hypothesis [16,17], but given that plastids are maternally inherited in ferns [52-54], plastid sequence data are insufficient for conclusive analyses of putative patterns of reticulation in polyploid complexes. Additional sampling of all taxa involved, and of additional, biparentally inherited nuclear markers, was clearly needed in order to resolve the relationships among the species in this group.

Here we present such an analysis for the North American reticulate complex of *Dryopteris*. Both previous studies that utilized nuclear sequence data [17,49] included only a single nuclear marker, so that evidence for the role of hybridization vs. introgression was lacking. In this study, we expanded sampling to include all taxa thought to be related to the reticulate complex based on previous analyses, and we present sequence data from the plastid genome and two nuclear markers, *pgiC* and *gapCp,* in most cases for multiple accessions*.* We use these data to unravel the history of the North American reticulate complex and to determine whether the sexual polyploids appear to have arisen via hybridization or introgression, whether such taxa have originated once or multiple times, and whether such origins require the existence of the missing diploid *“D. semicristata”.*

## Results

### Plastid phylogeny

The plastid dataset included 72 accessions representing 35 *Dryopteris* species, with two species of *Polystichum* used as outgroups (Table 2). The data matrix consisted of 7,913 aligned nucleotides, of which 1,825 (23%) were variable and 1,242 (16%) parsimony-informative under maximum parsimony (MP). Statistics for individual regions are given in Table 3. Indels provided an additional 254 characters, of which 104 (41%) were parsimony-informative within *Dryopteris*. Inclusion of indels in the MP analyses did not significantly alter topology, resolution, or clade support, so data were not included in subsequent maximum likelihood (ML) and Bayesian inference (BI) analyses, as the CIPRES Portal [55] does not provide a way to model standard (non-nucleotide) characters (see Methods). However, the MP results indicate that additional informative characters provided by the indel data likely would not have led to additional resolution or increased support values. Incongruence length difference (ILD) tests indicated significant conflict between the various regions of the plastid genome (*P* = 0.01). However, visual analysis of the phylogenies resulting from analyses of the various partitions did not reveal any discordance or conflict between well-supported clades, and so we proceeded with analysis of the combined dataset.

**Table 2:** **Accessions of *Dryopteris*** and ***Polystichum *included in this study**

**Species**	**Ploidy level (reference)**	**Collection locality**	**# *gapCp *copies**	**# *pgiC *copies**	**plastid?**	**Padre?**
*D. abbreviata*	2x [51]	Turkey	—	✓ (1)	✓	✓
*D. affinis*	2x, 3x [56]	Spain	✓ (2)	**	✓	—
*D. alpestris*	2x [57]	China	✓ (1)	✓ (1)	✓	✓
*D. antarctica*	unknown	Reunion	✓ (2)	✓ (3)	✓	—
*D. aquilinoides*	unknown	Reunion	✓ (2)	✓ (2)	✓	—
*D. arguta 1*	2x [58]	Oregon	✓ (1)	✓ (1)	✓	✓
*D. arguta 2*	2x [58]	Oregon	✓ (1)	✓ (1)	✓	—
*D. assimilis*^a^	2x [59]	Russia	—	✓ (1)	✓	✓
*D. austriaca*^b^	4x [40]	Caucasus region	—	✓ (1)	✓	—
*D. campyloptera 1*	4x [23]	North Carolina	✓ (2)	✓ (2)	✓	✓
*D. campyloptera 2*	4x [23]	North Carolina	✓ (2)	✓ (2)	✓	—
*D. carthusiana 1*	4x; [51]	New York	✓ (2)	✓ (2)	✓	✓
*D. carthusiana 2*	4x; [51]	Washington	✓ (2)	✓ (2)	✓	—
*D. carthusiana 3*	4x; [51]	Wisconsin	✓ (2)	✓ (2)	✓	—
*D. carthusiana 4*	4x; [51]	Wisconsin	—	✓ (2)	✓	—
*D. caucasica*	2x [35]	Turkey	✓ (2)	✓ (2)	✓	—
*D. celsa 1*	4x [24]	Georgia	✓ (1)	✓ (2)	✓	✓
*D. celsa 2*	4x [24]	South Carolina	✓ (2)	—	✓	—
*D. celsa 3*	4x [24]	Louisiana	✓ (2)	—	✓	—
*D. chrysocoma*	2x [60]	Taiwan	✓ (1)	✓ (3)	✓	—
*D. clintoniana 1*	6x [24]	New York	✓ (3)	✓ (3)	✓	✓
*D. clintoniana 2*	6x [24]	New York	✓ (3)	—	✓	—
*D. crispifolia*	4x [59]	BPSSE; W. Europe	—	—	✓	—
*D. cristata 1*	4x; [51]	South Carolina	✓ (2)	✓ (2)	✓	✓
*D. cristata 2*	4x; [51]	Iowa	✓ (2)	—	✓	—
*D. cristata 3*	4x; [51]	Pennsylvania	✓ (2)	—	✓	—
*D. cristata 4*	4x; [51]	Wisconsin	✓ (1)	—	✓	—
*D. cristata 5*	4x; [51]	New York	✓ (1)	—	✓	—
*D. cristata 6*	4x; [51]	Michigan	✓ (2)	—	✓	—
*D. cristata 7*	4x; [51]	Michigan	✓ (2)	—	✓	—
*D. dilatata 1*	4x; [51]	Germany	✓ (2)	✓ (2)	✓	—
*D. dilatata 2*	4x; [51]	Italy	✓ (1)	—	✓	—
*D. dilatata 3*	4x; [51]	France	—	✓ (2)	✓	—
*D. expansa 1*	2x [58]	British Columbia	✓ (1)	✓ (1)	✓	✓
*D. expansa 2*	2x [58]	Washington	✓ (1)	✓ (1)	✓	—
*D. expansa 3*	2x [58]	Oregon	✓ (1)	✓ (1)	✓	—
*D. expansa 4*	2x [58]	Washington	—	✓ (1)	✓	—
*D. filix-mas 1*	4x; [51]	British Columbia	✓ (2)	✓ (1)	✓	—
*D. filix-mas 2*	4x; [51]	Washington	✓ (2)	✓ (2)	✓	✓
*D. fragrans 1*	2x [58]	Michigan	✓ (1)	✓ (1)	✓	✓
*D. fragrans 2*	2x; [51]	Wisconsin	✓ (1)	✓ (1)	✓	—
*D. futura*	2x [61]	Guatemala	—	✓ (3)	✓	—
*D. goldiana 1*	2x [22]	North Carolina	✓ (1)	✓ (1)	✓	✓
*D. goldiana 2*	2x [22]	Wisconsin	✓ (1)	✓ (1)	✓	—
*D. goldiana 3*	2x [22]	New York	✓ (1)	✓ (1)	✓	—
*D. goldiana 4*	2x [22]	New York	✓ (1)	—	✓	—
*D. goldiana 5*	2x [22]	Michigan	✓ (1)	—	✓	—
*D. guanchica*	4x [59]	Cabildo of Tenerife	—	—	✓	—
*D. huberi*	unknown	Brazil	—	✓ (2)	✓	—
*D. intermedia 1*	2x [22]	North Carolina	✓ (1)	✓ (1)	✓	—
*D. intermedia 2*	2x [22]	Wisconsin	✓ (1)	✓ (1)	✓	✓
*D. intermedia 3*	2x [22]	New York	✓ (1)	✓ (1)	✓	—
*D. intermedia 4*	2x [22]	New York	✓ (1)	✓ (1)	✓	—
*D. intermedia 5*	2x [22]	New York	—	✓ (1)	✓	—
*D. intermedia 6*	2x [22]	Michigan	✓ (1)	—	✓	—
*D. ludoviciana A208*	2x [22]	South Carolina	—	*	—	—
*D. ludoviciana 1*	2x [22]	South Carolina	✓ (1)	✓ (1)	✓	✓
*D. ludoviciana 2*	2x [22]	Alabama	✓ (1)	✓ (1)	✓	—
*D. ludoviciana 3*	2x [22]	Alabama	—	✓ (1)	✓	—
*D. ludoviciana 4*	2x [22]	Alabama	—	✓ (1)	✓	—
*D. ludoviciana 5*	2x [22]	Alabama	✓ (1)	—	✓	—
*D. marginalis*	2x [58]	South Carolina	✓ (1)	✓ (1)	✓	✓
*D. monticola*	unknown	Japan	—	✓ (2)	✓	—
*D. muenchii 1*	3x [62]	Mexico	✓ (3)	✓ (3)	✓	—
*D. muenchii 2*	3x [62]	Mexico	✓ (3)	✓ (3)	✓	—
*D. oligodonta*	2x [59]	Cabildo of Tenerife	—	✓ (1)	✓	✓
*D. oreades*	2x [63]	Caucasus region	—	**	✓	✓
*D. pallida*	2x [58]	AFSSE; W. Europe	—	✓ (1)	✓	✓
*D. remota 1*	3x; [51]	Germany	✓ (2)	—	✓	—
*D. remota 2*	3x; [51]	Asia	—	✓ (2)	✓	—
*D. scottii*	4x [60]	Taiwan	—	✓ (2)	✓	—
*D. tokyoensis*	2x [58]	Japan	✓ (1)	✓ (1)	✓	✓
*Polystichum andersonii*		Washington	—	✓ (1)	✓	—
*Polystichum munitum*		Washington	✓ (1)	—	✓	✓

Numbers after species names indicate that multiple accessions of that species were included. Collection locations are given, and inclusion in plastid, *pgiC, gapCp*, and PADRE datasets is indicated. AFSSE and BPSSE indicate species that were obtained as spores from the American Fern Society Spore Exchange and British Pteridological Society Spore Exchange, respectively. These spores were germinated and grown for use by Geiger and Ranker [64], and DNA material later provided to us. Number of copies of *gapCp* and *pgiC* are given in parentheses if we successfully sequenced that region for an accession. Ploidy level is given when known, with references. See Additional file 1: Table S1 for voucher information.

* The *pgiC* sequence for *D. ludoviciana* A208 was obtained from Genbank, and we included it in the current study as it was the basis for a recent rejection of the “semicristata” hypothesis [49].

** We were unable to sequence *pgiC* from our *D. affinis* and *D. oreades* accessions, and so obtained *pgiC* sequences for these taxa from Genbank. These sequences are therefore not from the same accessions as the *gapCp* and plastid sequences.

^a^ synonymous with *D. expansa*.

^b^ synonymous with *D. dilatata*.

**Table 3:** Statistics for the plastid and nuclear genomic regions sequenced for this study

				**With outgroup**		**Just *Dryopteris***		**Indels**	
**Region**	**Primer source**	**Aligned bases**	**Optimal model of evolution**	**Variable bases**	PIC*	**Variable bases**	PIC*	**#, just in *Dryopteris***	**PIC**
*rbcL*	Korall et al., 2006 [65]	1365	GTR+Γ	179 (13%)	116 (8%)	163 (12%)	93 (7%)	34	16 (47%)
*rbcL-accD*	Korall et al., 2007 [66]	1650	GTR+I+Γ	261 (16%)	161 (10%)	226 (14%)	123 (7%)	37	7 (19%)
*trnG-trnR*	Korall et al., 2007 [66]	1057	HKY+Γ	277 (26%)	204 (19%)	229 (22%)	165 (16%)	34	14 (41%)
*psbA-trnH*	Kress et al., 2005 [67]	476	HKY	75 (16%)	48 (10%)	62 (13%)	35 (7%)	11	3 (27%)
*trnP-petG*	Small et al., 2005 [68]	551	GTR+Γ	195 (35%)	142 (26%)	166 (30%)	115 (21%)	30	18 (60%)
*rps4-trnS*	Rouhan et al., 2004 [69]	464	HKY+Γ	160 (34%)	112 (24%)	125 (27%)	84 (18%)	17	4 (24%)
*trnL-F*	Taberlet et al., 1991 [70]	352	HKY+Γ	92 (26%)	59 (17%)	70 (20%)	41 (12%)	12	5 (42%)
*matK*	Duffy et al., 2009 [71]	977	HKY+Γ	250 (26%)	189 (19%)	189 (19%)	133 (14%)	12	2 (17%)
*trnV-trnM*	Small et al., 2005 [68]	1021	HKY+Γ	336 (33%)	211 (21%)	271 (27%)	154 (15%)	67	35 (52%)
Total plastid	---	7913	---	1825 (23%)	1242 (16%)	1501 (19%)	943 (12%)	254	104 (41%)
*pgiC*	Ishikawa et al., 2002 [72]	744	HKY+Γ	163 (22%)	97 (13%)	137 (18%)	96 (13%)	35	20 (57%)
*gapCp*	Schuettpelz et al., 2008 [73]	729	GTR+Γ	229 (31%)	144 (20%)	197 (27%)	141 (19%)	62	34 (55%)

^*^Parsimony-informative characters.

**Figure 2:**
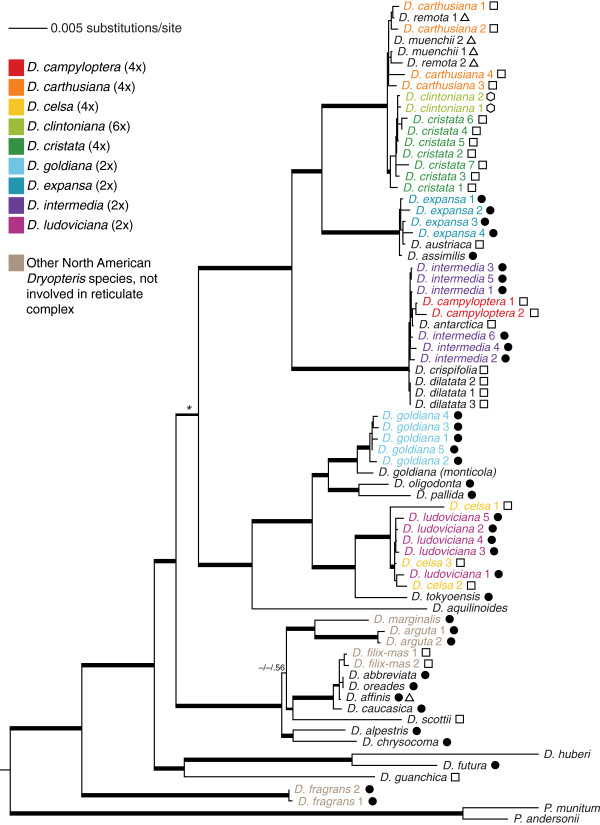
**Best maximum likelihood topology from analysis of the plastid dataset.** Thickest lines indicate strong support (MP BS ≥ 70%, ML BS ≥ 70% *and* BI PP ≥ 95%), medium lines indicate moderate support (either ML BS ≥ 70% *or* BI PP ≥ 95%), and thin lines indicate weak support (ML BS ≤ 70% *and* BI PP ≤ 95%). * indicates a node along the backbone of the phylogeny that received support only from BI analysis. Support values are given as MP BS/ML BS/BI PP. The North American species are colored according to the legend given. Numbers after taxon names indicate multiple accessions of that species (see Table 2). Symbols denote ploidy: solid circles are diploids, triangles triploids, squares tetraploids, and hexagons the hexaploid *D. clintoniana*.

MP analysis identified 185 most-parsimonious trees of length 2817 steps, with CI = 0.72 and CI’ = 0.63. ML analysis in Garli produced a single most likely tree with -ln 27272.94 (Figure 2), and MP bootstrap, ML bootstrap, and BI analyses produced highly congruent consensus topologies that were moderately well resolved (29, 43, and 45 of 72 nodes resolved, respectively; unresolved nodes were concentrated at the tips of the trees, and comprised multiple accessions of one or more species). The backbone of the phylogeny was highly resolved and strongly supported in all analyses, except for one node (indicated with an asterisk in Figure 2), which received strong support only from BI analysis (MP bootstrap/ML bootstrap/BI posterior probability = 65/67/.96). Two accessions of *Dryopteris fragrans* were strongly supported as sister to each other, and together sister to the rest of *Dryopteris*. For those species for which multiple accessions were included, sequences for all accessions fell into the same clade, though in several cases accessions from multiple species grouped together with strong support (e.g. *D. clintoniana* and *D. cristata*).

### *pgiC* phylogeny

The *pgiC* dataset included 55 accessions representing 33 *Dryopteris* species and one species of *Polystichum* (Tables 2 and 3). The data matrix consisted of 744 aligned nucleotides, of which 163 (22%) were variable and 97 (13%) parsimony-informative under MP. As with the plastid dataset, indel data did not significantly increase resolution or clade support, and these data were not included in the ML and BI analyses performed in CIPRES. The number of *pgiC* copies found per species agreed well with the known ploidy for most taxa, and for species with more than copy we consider the copies to be homeologs. Of the 55 *Dryopteris* accessions in the *pgiC* dataset, 30 were from species known to be diploids (Table 2), and 27 of these had one *pgiC* copy. Diploid *D. caucasica, D. chrysocoma,* and *D. futura* were found to have two, three, and three copies, respectively. Fifteen accessions were of tetraploid taxa, and all but three of these had two *pgiC* copies; *D. austriaca* and one accession of *D. filix-mas* each had one, and *D. antarctica* had three. Four accessions in the *pgiC* dataset were either triploid or hexaploid species, and these each had three copies, except for *D. remota*, which had two.

**Figure 3:**
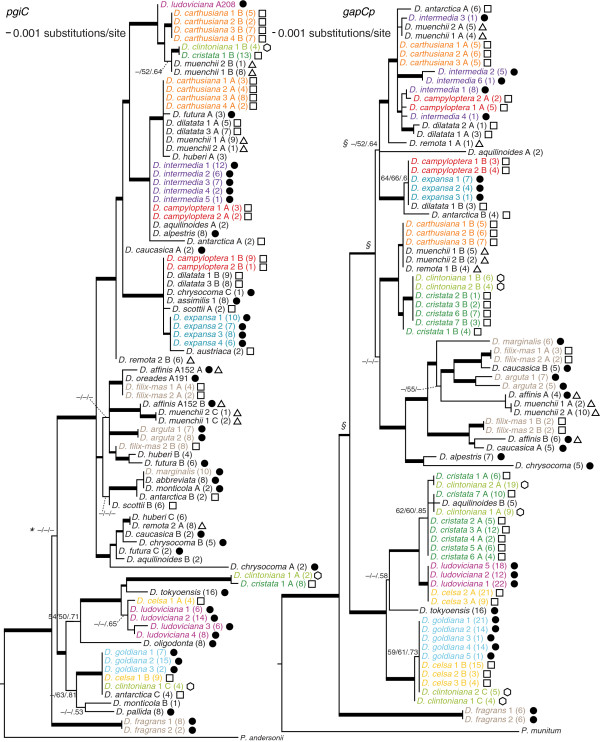
**Best maximum likelihood topologies from analyses of *pgiC *and *gapCp*.** Colors, symbols, and line weights indicating support for clades as in Figure 2. A, B, and C following taxon names indicate that for a given accession we found multiple copies of that marker. Parentheses enclose the number of clones whose sequences are represented by each consensus allele sequence. * and Â§ indicate nodes along the backbone in the *pgiC* and *gapCp* topologies, respectively, that were not highly supported in our analyses.

MP analysis of the *pgiC* matrix identified 558 most-parsimonious trees of length 220 steps, with CI = 0.82 and CI’ = 0.74. ML analysis produced a single best tree with -ln 2433.50 (Figure 3), and MP bootstrap, ML bootstrap, and BI analyses produced highly congruent but poorly resolved consensus topologies: 15, 40, and 25 of 86 nodes resolved, respectively. As with the plastid phylogeny, much of the lack of resolution involved multiple accessions of one or more species (e.g. the clades containing *D. carthusiana, D. intermedia, D. campyloptera,* and *D. expansa* in Figure 3). The backbone generally received strong support, except for one node (indicated with an asterisk in Figure 3) which resolved *D. fragrans* as sister to the rest of the genus, with the latter being split into two well-resolved clades comprising *D. goldiana, D. ludoviciana*, and their relatives vs. all other species. Although *D. fragrans* was resolved as sister to the rest of *Dryopteris* in the best ML topology (and in the plastid analyses), its relationship to the *D.goldiana-D. ludoviciana* and “all others” clades was ambiguous in all other *pgiC* analyses, resulting in the lack of support and resolution at this node. In addition, the position of the clade containing *D. arguta, D. filix-mas,* and *D. marginalis* was unresolved relative to two other clades containing, respectively, *D. remota* plus several related taxa, and a large clade containing *D. intermedia* and *D. expansa* as well as several North American allopolyploids and taxa from other regions.

For species represented by multiple accessions, sequences from all accessions fell into the same clade, and for taxa with multiple copies of *pgiC,* separate, well-supported clades generally formed that contained the various copies. A sequence of *D. ludoviciana* obtained from Genbank, that was the basis for a recent rejection of the “semicristata” hypothesis [49], was uniquely resolved as sister to a clade containing sequences of *D. carthusiana, D. clintoniana, D. cristata,* and *D. muenchii,* as found by Juslen et al. [49]. This placement was different from that of our four accessions of *D. ludoviciana*, which fell together in a strongly supported clade that also contained *D. tokyoensis* and several North American allopolyploids (Figure 3), congruent with *D. ludoviciana*’s placement in our plastid phylogeny.

### *gapCp* phylogeny

The *gapCp* dataset included 52 accessions representing 23 species of *Dryopteris* and one of *Polystichum* (Tables 2 and 3). The data matrix consisted of 729 aligned nucleotides, of which 229 (31%) were variable and 144 (19%) parsimony-informative under MP. As with the plastid and *pgiC* datasets, indel data did not significantly increase resolution or clade support for *gapCp*, and these data were not included in the ML and BI analyses performed in CIPRES.

As with *pgiC,* the number of *gapCp* copies found agreed well with the known ploidy of most taxa, and for species with more than *gapCp* copy we consider the copies to be homeologs. Of the 52 *Dryopteris* accessions analyzed, 26 were from known diploid species (Table 2), and 23 of these had one *gapCp* copy. *D. affinis, D. caucasica,* and *D. chrysocoma* were found to have two, three, and two copies, respectively. Twenty accessions were of tetraploid taxa, and all but four had two *gapCp* copies: for one accession each of *D. celsa* and *D. dilatata*, and two of *D. cristata*, we found only one copy of *gapCp.* Five accessions were either triploid or hexaploid taxa, and all except *D. remota* had three *gapCp* copies; as with *pgiC,* we only found two copies in *D. remota*.

MP analysis of the *gapCp* matrix identified 478 most-parsimonious trees of length 358 steps, with CI = 0.73 and CI’ = 0.64. ML analysis produced a single best tree with -ln 3090.94 (Figure 3), and MP bootstrap, ML bootstrap, and BI analyses produced highly congruent but poorly resolved consensus topologies, with 22, 37, and 31 of 82 nodes resolved, respectively. As with the plastid and *pgiC* phylogenies, much of the lack of resolution occurred where multiple accessions of one or more species were concentrated, but several nodes along the backbone received lower support than in analyses based on the other markers (indicated with Â§ in Figure 3). *D. fragrans* was resolved as sister to the rest of the genus, though with only moderate support (MP bootstrap/ML bootstrap/BI posterior probability = 67/74/.55), and within the rest of *Dryopteris*, relationships between several large clades were generally congruent between analyses but lacking support. The clade containing *D. arguta, D. filix-mas,* and *D. marginalis* was resolved in the best ML topology as sister to a clade containing sequences of several polyploid taxa, but this relationship did not receive support from the other analyses. As with *pgiC,* sequences of species represented by multiple accessions fell into the same clade, and for taxa with multiple copies of *gapCp,* separate, well-supported clades generally formed that contained the various copies, though often with additional species present.

### Divergence time analysis

After 35,000,000 generations, all effective sample size (ESS) values for the divergence time analysis (as viewed in Tracer) were well above the recommended threshold of 200, indicating that parameter space had been sufficiently sampled. The coefficient of variation indicated that the data were not evolving in a clock-like fashion (value above 0.5), and the uncorrelated lognormal (UCLN) model was thus the most appropriate model of rate variation for this set of loci. Cladogenetic events within *Dryopteris* were estimated as beginning ca. 42 Ma ( 4), with the divergence between the ancestors of *D. fragrans* and the rest of the genus. The North American allopolyploids in the reticulate complex diverged from their closest living relatives within the last ca. 7 Ma (Table 4). A clade containing the shared copies of *D. carthusiana, D. cristata,* and *D. clintoniana* diverged from its closest diploid relatives ca. 26 Ma. *D. filix-mas’s* copies diverged from their sister taxa less than ca. 10 Ma.

**Figure 4:**
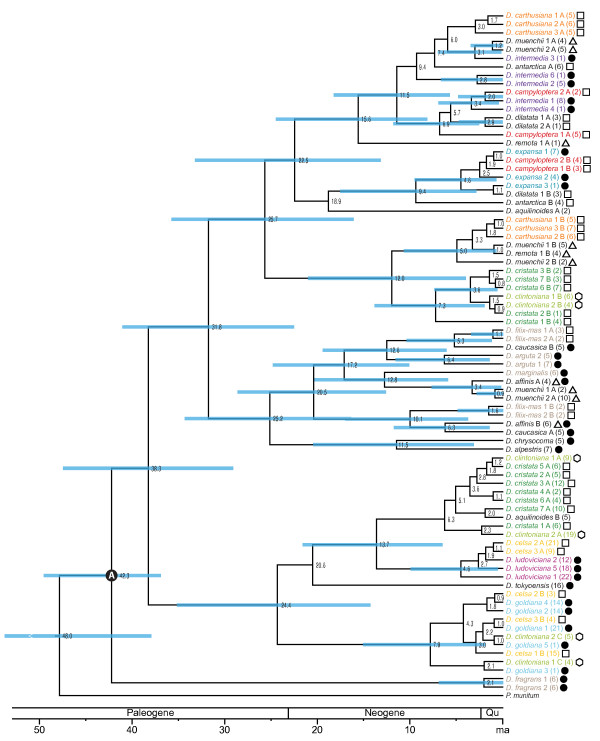
**Maximum clade credibility chronogram from BEAST analysis of *gapCp.*** Mean divergence time estimates are given, and blue bars represent 95% highest posterior density (HPD) intervals around these means. Branches without bars were present in fewer than 50% of trees in the posterior distribution and so did not receive annotation. Black circle with A indicates the node used for calibration, which was modeled as a lognormal prior based on a secondary estimate of the root age of *Dryopteris*[16]. Colors are as in Figures 2, 3.

**Table 4:** Inferred ages of North American allopolyploid formation

**Allopolyploid**	**Oldest divergence from putative maternal parent**	**Oldest divergence from putative paternal parent**	**Inferred age of earliest polyploid formation**
*D. campyloptera* (4x)	*D. intermedia* (2x), 6.9 Ma	*D. expansa* (2x) 4.6 Ma	≤ 4.6 Ma
*D. carthusiana* (4x)	*“D. semicristata”* (2x?), 25.7 Ma	*D. intermedia* (2x), 11.5 Ma	≤ 11.5 Ma
*D. celsa* (4x)	*D. ludoviciana* (2x), 4.6 Ma	*D. goldiana* (2x), 7.9 Ma	≤ 4.6 Ma
*D. clintoniana* (6x)	*D. cristata* (4x), 13.7 and 7.3 Ma	*D. goldiana* (2x), 7.9 Ma	≤ 7.3 Ma
*D. cristata* (4x)	*“D. semicristata”* (2x?), 25.7 Ma	*D. ludoviciana* (2x), 13.7 Ma	≤ 13.7 Ma
*D. filix-Mas* (4x)	*D. oreades/D. abbreviata/D. affinis/D. caucasica,* 5.3 Ma	*D. affinis/D. caucasica?*, 10.1 Ma	≤ 5.3 Ma

The dates of divergence between the allopolyploid homeologs and their closest diploid relatives are given. The age of earliest formation for each polyploid is inferred to be the younger of these two dates.

### Reticulation network

**Figure 5:**
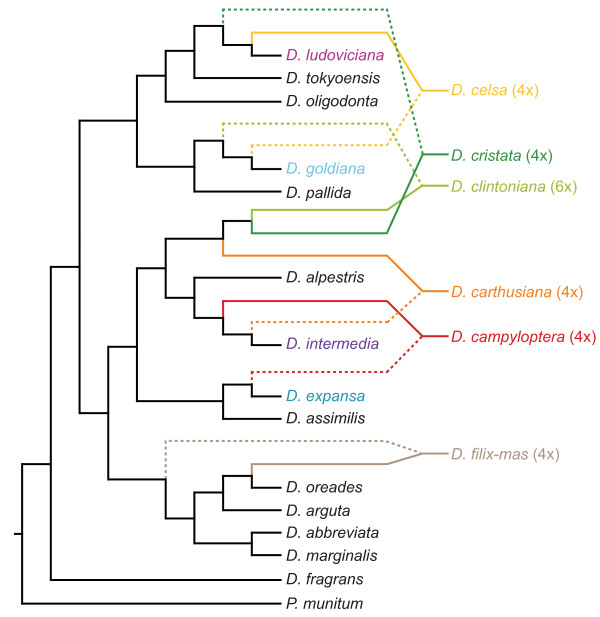
**Reticulation network showing hypothesized polyploidization events.** Redrawn from the PADRE analysis of combined *pgiC* and *gapCp* dataset for North American allopolyploids and all other non-reticulate taxa included in the current study. Ploidy is indicated for allopolyploids; all other taxa are diploid (2x; see Table 2). Solid lines indicate the plastid lineage. Dotted lines indicate the paternal lineage as determined from nuclear sequence data. Taxa are colored as in Figures 1, 2 and 3.

**Figure 6:**
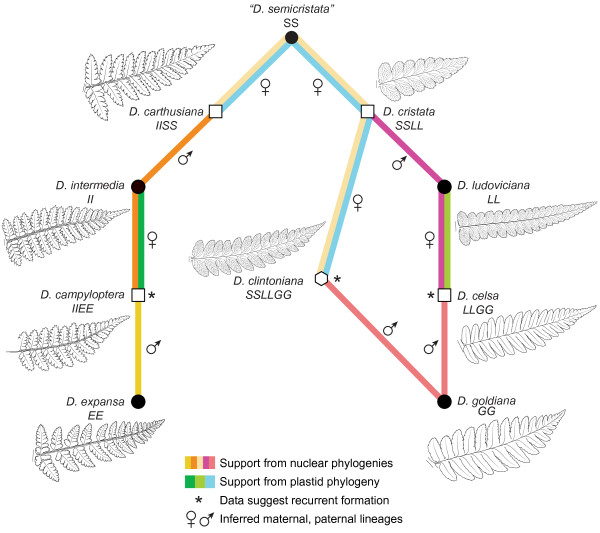
**Summary of plastid and nuclear sequence support for parentage of the allopolyploids in the North American *Dryopteris *complex.** Colored lines connect diploids progenitors with allopolyploid offspring according to the “semicristata” hypothesis [12,14]. Warm colors (yellows, oranges, and pinks) denote support from the nuclear phylogenies, and cool colors (greens and blue) denote support from the plastid phylogeny. Maternal and paternal lineages are indicated by symbols. Outlines of pinnae from each species are given for comparison. Fraser-Jenkins [74] and Stein et al. [48] (based on work by Werth and Kuhn [45]) have produced reconstructions of *D. semicristata* that depict it as more similar to *D. cristata* or *D. carthusiana*, respectively.

An ILD test was performed on the data matrix used to generate the reticulation network, and it indicated no significant conflict between *gapCp* and *pgiC* for the 20 *Dryopteris* species using to conduct the reticulation analysis (*P* = 0.1). The network produced by PADRE identified six genome merger or allopolyploidization events, which correspond to the six allopolyploid species present in North America (Figure 5). For *D. filix-mas*, *D. oreades* was identified as one progenitor, but the second genome could not be assigned to any of the taxa included. For the five polyploid members of the reticulate complex, the allopolyploidization events combined genomes of the inferred progenitor taxa as predicted by the “semicristata” hypothesis. *D. campyloptera*’s two genomes were assigned to *D. expansa* and *D. intermedia*, and *D. celsa*’s to *D. goldiana* and *D. ludoviciana*. Two of *D. clintoniana*’s three genomes were assigned to *D. cristata*, and the third to *D. goldiana*. The three polyploids putatively descended from *“D. semicristata”, D. clintoniana, D. cristata,* and *D. carthusiana,* shared one genome in common that was not assignable to any single extant diploid taxon. Support for the various relationships from our plastid, nuclear, and reticulation analyses is summarized and superimposed on a representation of the “semicristata” hypothesis in Figure 6.

### Genetic distances

**Figure 7:**
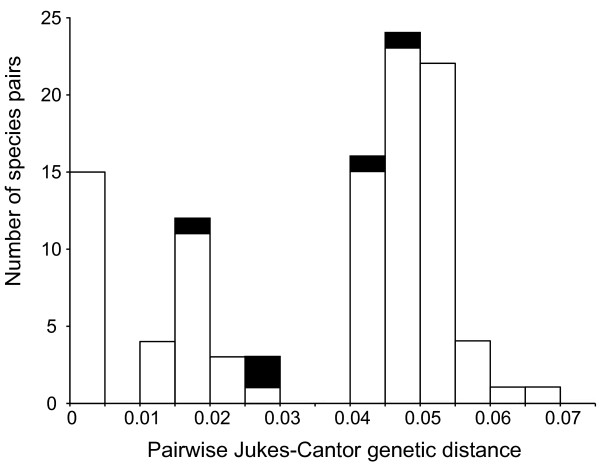
**Histogram of pairwise Jukes-Cantor distances between all diploid species pairs.** Parental pairs of the five allopolyploids are shown in black.

A histogram of Jukes-Cantor distances based on plastid data for all pairs of diploid species is shown in Figure 7. The pairs of species corresponding to the actual parents of the allopolyploids (shown in black) rank 28, 36, 37, 39, and 70 out of 105, and exhibit an intermediate degree of genetic divergence compared with all potential pairs of diploid parents (*P* = 0.017).

## Discussion

### Allopolyploid origins in the north American reticulate complex

Ever since the first cytological evaluations of the allopolyploid taxa in this complex [13,21,51], various lines of evidence have stimulated the development of numerous hypotheses to explain these species’ relationships and parentage (Table 1, Figure 1). Our analyses, which are based on the most extensive sampling of North American *Dryopteris* taxa and loci to date, unambiguously support the “semicristata” hypothesis as an explanation for this group’s evolutionary history. This hypothesis proposes that *D. campyloptera* is an allotetraploid hybrid between *D. expansa* and *D. intermedia*; *D. celsa* is an allotetraploid hybrid between *D. goldiana* and *D. ludoviciana*; *D. clintoniana* is an allohexaploid hybrid between *D. ludoviciana* and tetraploid *D. cristata*; and *D. carthusiana* and *D. cristata* are allotetraploid hybrids with one extinct parent in common (*“D. semicristata”*), and *D. intermedia* and *D. ludoviciana*, respectively, as their second parents (Figure 1A). Our results are congruent with all aspects of this hypothesis (Figure 6), and conflict directly with one or more predictions of each of the competing hypotheses. Despite the interest in this group historically, DNA sequence data were not brought to bear on this question until relatively recently. Sessa et al. [16] produced a phylogeny based on seven plastid markers that supported the “semicristata” hypothesis, though analysis of a uniparentally inherited marker was insufficient for identifying both parents of the hybrids. Juslen et al. [49] conducted the first phylogenetic analysis of the group based on nuclear sequence data, and rejected the “semicristata” hypothesis based on the placement of a single accession of *D. ludoviciana*. Its location in their phylogeny suggested that *D. ludoviciana* was in fact the missing shared parent of *D. cristata* and *D. carthusiana*.

The key difference between the “semicristata” hypothesis and competing explanations for the group’s history is the putative parentage of *D. cristata* and *D. carthusiana*. Early cytological analyses of artificial crosses between species (described above) revealed that these two allopolyploids share a genome in common, and the identity of this missing parent is the focal point of debate. The “semicristata” hypothesis [13,14] posits an extinct species in this role, while other theories have focused on either *D. ludoviciana*[37,40,41] (Figure 1B, C, E) or *D. tokyoensis*[43] (Figure 1D), based on alternative explanations of cytological observations and chromatographic analyses. Fraser-Jenkins [74], in reviewing the various studies on the group, rejected each of these species as the missing progenitor based on additional studies and morphological features, but the idea of *D. ludoviciana*’s involvement persists, as demonstrated by Juslen et al.’s [49] recent work. The key to untangling this conundrum rests on whether genomes of the three putative descendants of *“D. semicristata”* can be assigned unequivocally to an extant diploid species (viz., *D. ludoviciana, D. tokyoensis*, or another taxon); based on our findings, they cannot.

The analyses conducted in the current study unequivocally support the “semicristata” hypothesis and the existence of the missing diploid species. Our plastid data set greatly expands taxon sampling compared to the plastid-based analyses of Sessa et al. [16] and Juslen et al. [49] (who included a phylogeny based on *trnL-F* in their study) by including multiple individuals of each North American species collected from across their geographic ranges in North America (Table 2, Figure 7). Sequences from each of the allopolyploids grouped together in the plastid phylogeny, with the three putative offspring of *“D. semicristata”* placed together, with strong support, in a clade that also included two apomictic triploid species, *D. muenchii* and *D. remota*, but no diploids. The plastid data therefore indicate that these taxa share a maternally-donated genome, with *D. cristata* having been the maternal donor to *D. clintoniana*; these two species form a subclade, and the former is predicted to be one parent of the latter in all hypotheses (Table 1). Of the competing explanations, only the “semicristata” scheme would predict these three allopolyploids’ placement in a clade with no additional, extant diploids. The genome they share is, according to all other hypotheses, supposed to have been donated either by *D. ludoviciana*[37,40,41] or *D. tokyoensis*[43], but these two species are distantly located in our plastid phylogeny. The two additional allotetraploids in the reticulate complex, *D. celsa* and *D. campyloptera*, were each strongly supported in our plastid analyses as sister to one of their putative parents as predicted by the “semicristata” hypothesis: *D. ludoviciana* for *D. celsa*, and *D. intermedia* for *D. campyloptera* (Figure 6). Both of these relationships are at odds with early explanations for the two tetraploids’ parentage [20,29], but are congruent with more recent hypotheses, including the “semicristata” scheme and others. The *D. intermedia–D. campyloptera* clade also includes several Eurasian allopolyploids, several of which have been suggested to be additional carriers of the *D. intermedia* genome [44,75].

For *pgiC* and *gapCp,* the expected number of gene copies was found for each of the North American species based on their ploidy (Table 2): diploids had one copy of each marker, tetraploids had two, and the hexaploid *D. clintoniana* had three, although for three tetraploid accessions (two of *D. cristata* and one of *D. celsa*), we were only able to isolate one. We consider copies of *pgiC* and *gapCp* to be homeologs if a given species has more than one copy for a given locus. Because all species that contained multiple copies of the nuclear loci have been thoroughly documented in the past as being polyploids, and because these species possessed multiple copies of both loci while none of the known diploids did, we are confident that the multiple copies represent homeologs and not just allelic diversity at each of the loci. The *pgiC* and *gapCp* phylogenies (Figure 3) concur with the plastid phylogeny on the identity of one parent of each of the allopolyploids (the maternal progenitor). Sets of homeologs from *D. carthusiana, D. clintoniana,* and *D. cristata* formed well-supported clades in both topologies that also included *D. muenchii* and/or *D. remota*, but no diploid taxa (except the Juslen et al. [49]*D. ludoviciana* sequence, discussed below). This suggests that these latter two species are additional descendants of *“D. semicristata”*; they cannot be “*D. semicristata*” because the ploidy levels of the North American allotetraploids require the missing taxon to be a diploid, which *D. muenchii* and *D. remota* are not (Table 2) [62,76].

The second set of homeologs from each of the allotetraploids grouped with their second proposed parent: *D. carthusiana* with *D. intermedia*, and *D. cristata* with *D. ludoviciana*. *D. clintoniana*’s two additional homeologs fell with its inferred paternal progenitor, *D. goldiana*, and with the paternal copies of its putative mother, *D. cristata*, and *D. cristata*’s putative father, *D. ludoviciana*. This overall pattern is congruent only with the “semicristata” hypothesis. As mentioned above, other hypotheses predict that *D. carthusiana* should have copies of nuclear markers that are closely related to *D. ludoviciana* or *D. tokyoensis*. Instead, homeologs from *D. carthusiana, D. cristata,* and *D. clintoniana* fall into a clade without any extant diploid species, as in the plastid phylogeny. Our reticulation network demonstrates this as well, with the shared genome from these three taxa not assigned to a diploid species (Figure 5).

One alternative explanation for *D. cristata*’s origin has *D. goldiana* as one of its proposed parents [37] (Figure 1B), and this is also not supported by our analyses, as *D. cristata* has no homeologs that are closely related to *D. goldiana*. We also reject the hypothesis that *D. tokyoensis* is the missing ancestor [43] (Figure 1D), as the shared genome is clearly not closely related to *D. tokyoensis* in any of our phylogenies. However, the *pgiC* phylogeny did place *D. cristata* and *D. clintoniana* as more closely related to *D. tokyoensis* than to *D. ludoviciana*, though only with moderate support (Figure 3). *D. tokyoensis* and *D. ludoviciana* are known to be quite closely related, however [43,77], and given the short branch placing the polyploids with *D. tokyoensis*, it seems more likely that incomplete lineage sorting of this locus between the two closely related diploids is responsible for the observed relationship. The best ML topology used to produce the reticulation network was based on a combined analysis of *gapCp* and *pgiC,* and *D. cristata* was more closely related to *D. ludoviciana* than to *D. tokyoensis* in this tree (Figure 5). We also note that one copy of *D. aquilinoides* appears to be closely related to *D. cristata* based on our *gapCp* topologies (Figures 3, 4). This species’ ploidy is unknown, but we infer that it is a tetraploid based on its possession of two copies each of *gapCp* and *pgiC.* It may have *D. ludoviciana* or *D. goldiana* as one parental species, based on its position in the *gapCp* and plastid phylogenies (Figures 2, 3 and 4).

In addition to providing evidence for the putative descendants of “*D. semicristata”*, the *gapCp* and *pgiC* phylogenies also fully support the hypothesized parentage of *D. celsa* and *D. campyloptera* (Figure 6). The results of the plastid phylogeny are confirmed, with *D. intermedia* again strongly supported as one parent of *D. campyloptera*, and *D. ludoviciana* of *D. celsa*, and the nuclear phylogenies add *D. expansa* and *D. goldiana* as the second parents of each, respectively, with moderate to strong support for all relationships from both analyses. These relationships have not been as contentious historically as the origins of the *“D. semicristata”* descendants, but our analyses provide the first unequivocal evidence from DNA sequence data in support of these species proposed origins. Our data also support an allotetraploid origin for *D. dilatata* (= *D. austriaca*), which has long been thought to represent a cross between *D. intermedia* and *D. expansa*[13,40,59,75,78], making it the European equivalent of *D. campyloptera* in North America. Such a history is congruent with the analyses presented here.

We sequenced *pgiC* from four accessions of *D. ludoviciana* for this study, and also included the *D. ludoviciana pgiC* sequenced produced by Juslen et al. [49] that was the basis for their rejection of the “semicristata” hypothesis. As in their study and that of Sessa et al. [17], this single sequence was placed sister to the clade containing homeologs from *D. carthusiana, D. cristata,* and *D. clintoniana*, and it was this placement that led them to claim *D. ludoviciana* as the source of the shared genome*.* The position of this sequence is totally different from that of the four new *D. ludoviciana* accessions included here. The congruence in position of multiple accessions of this species in the current study strongly supports the suggestion of Sessa et al. [17] that some type of contamination or PCR error may have been involved in the generation of the Juslen et al. sequence; such errors are common in cloning-based studies of single and low-copy nuclear markers [79], and rejection of a long-standing hypothesis based on a single sequence seems unwise when such errors are possible. Juslen et al. [49] did not include *D. ludoviciana* in their *trnL-F* phylogeny, which would have allowed an independent assessment of that accession’s phylogenetic position based on an unlinked marker. The plastid and *gapCp* phylogenies presented in the current study are completely congruent with the *pgiC* phylogeny on the placement of multiple *D. ludoviciana* accessions, further supporting our contention that the Juslen et al. sequence is the result of error and should not be considered grounds for rejection of the “semicristata” hypothesis.

The addition of a second, unlinked nuclear marker in the current study also allows us, for the first time, to assess the potential role of introgression in shaping the relationships among these taxa. Introgression following polyploidization would be unlikely once a ploidy barrier had been established between allopolyploids and their progenitors [80], and the congruence in the positions of the allopolyploid homeologs in our *gapCp* and *pgiC* phylogenies strongly supports whole genome merger (i.e. allopolyploidization) rather than introgression. The latter would not be expected to produce the identical patterns in unlinked markers [81] that we observe here. Previous studies of isozymes [45,46,82] and chromatography [42] that demonstrated additivity of numerous compounds in the North American allotetraploids are also consistent with whole-genome merger rather than isolated incidents of introgression in the immediate histories of the hybrids. However, the lack of support along the backbones of our *pgiC* and *gapCp* phylogenies, and incongruence between them in the placement of several clades, suggests that deeper coalescent processes – such as ancient hybridization, introgression, or incomplete lineage sorting – may have played a role during the evolution of *Dryopteris* as a whole. In the *pgiC* phylogeny, the clade containing *D. intermedia* and the A homeologs of *D. carthusiana* and *D. campyloptera* is sister to the clade containing the B homeologs of *D. carthusiana, D. clintoniana* and *D. cristata* (the “semicristata” clade); these two together are sister to a clade containing *D. expansa*, suggesting that *D. intermedia* is *“D. semicristata”*’s closest living diploid relative. Analyses of *pgiC* for a somewhat different sampling of *Dryopteris* taxa by Sessa et al. [17] indicated the same. In the best *gapCp* ML phylogeny, the *D. intermedia/D. carthusiana* A clade is sister, with low support, to the *D. expansa* clade, with the “semicristata” clade more closely related (though with no support) to a clade containing *D. marginalis*, *D. arguta*, and *D. filix-mas*, among others. This latter clade’s placement was unresolved in the *pgiC* topology. In the Bayesian analysis of *gapCp* (represented by the chronogram, Figure 4), the “semicristata” clade is equally closely related to the *D. intermedia* and *D. expansa* clades, and the *D. filix-mas* clade is sister to all of them. The plastid data from the current study and Sessa et al. [17] strongly support *D. expansa* as the closest living relative of *“D. semicristata”*. These incongruences between loci and analyses may reflect one or more of the coalescent phenomena mentioned above. Phylogenetic and concordance analyses [83,84] preferably of dozens of nuclear markers to determine the dominant history of the nuclear genome should be the next step in assessing relationships among the diploids in this group and determining whether the closest living relative of *“D semicristata”* is *D. intermedia* or *D. expansa*.

### *Dryopteris filix-mas*

Although *D. filix-mas* was not the primary focus of the current study, as it has not played an active role in the North American reticulate complex, its origins have long attracted the attention of systematists and our sequence data may be able to contribute somewhat to the elucidation of its history. It is generally thought to have formed via hybridization and subsequent polyploidization between two separate Eurasian species. The most commonly cited are *D. abbreviata* and *D. caucasica*[19,38], and the currently accepted hypothesis has *D. filix-mas* as an allotetraploid hybrid of the two (Table 1, Figure 1). There is some taxonomic confusion with regard to these species and other potential progenitor taxa, however, which hinders comprehension of this group [30]. The name *D. oreades* was cited by Fraser-Jenkins [85] as replacing the name *D. abbreviata*, but they are not currently accepted as synonyms for each other, and *D. abbreviata* is instead an accepted synonym of *D. pseudomas*. We included one accession each of *D. caucasica, D. abbreviata,* and *D. oreades*, but were unable to amplify *gapCp* from *D. abbreviata*, or either *pgiC* or *gapCp* from *D. oreades* (a *pgiC* sequence obtained from GenBank was included for *D. oreades* in the current study). Despite these limitations, our analyses do support a role for *D. oreades/D. abbreviata*, as well as *D. caucasica*, in D. *filix-mas*’s origins. These species fell together in the plastid phylogeny (Figure 2), along with *D. affinis*, and the latter also appeared to be closely related to *D. filix-mas* in both the *gapCp* and *pgiC* topologies. *D. affinis* is known to have both diploid and triploid forms [56], and may have played some role in the formation of *D. filix-mas.* Based on these analyses we cannot reject the current hypothesis for this species’ origin; neither can we fully accept it, and the role of *D. affinis* in particular deserves further study.

One additional complication in understanding *D. filix-mas*’s history centers on whether the forms of this taxon in North America and Europe are the same. It has been suggested that they are separate evolutionary lineages [19], and even that eastern and western forms in North America merit separate consideration [33]. The two accessions included here were both collected from western North America, and the next step in understanding *D. filix-mas*’s origins should begin with thorough sampling of this taxon and all possible progenitors throughout their ranges in North America and Eurasia.

### Timing and recurrence of polyploidization events

Recurrent formation of a polyploid occurs when a given species arises repeatedly from separate crosses between different individuals of the same set of parental taxa. This phenomenon is now recognized as prevalent in the evolutionary histories of most polyploid lineages [86,87], and has been demonstrated for several fern groups, including *Asplenium*[88,89], *Polystichum*[54], *Astrolepis*[90], and *Dryopteris* (C. Werth, unpublished data, cited by [91]). Recurrent origins can be inferred when genetic material from different accessions of a polyploid are more closely related to separate individuals from one or more of the parental taxa [86], though introgression of markers via backcrossing with a progenitor can also lead to multiple genotypes within a polyploid lineage that has had only a single origin [92]. Changes will also accumulate in DNA subsequent to polyploidization due to natural microevolutionary processes, and sequences are thus not expected to be identical between polyploids and progenitors, particularly in more ancient polyploids [93].

In *Dryopteris*, Soltis and Soltis [91] cite unpublished isozyme analyses conducted by the late Charles Werth that supported multiple origins of *D. campyloptera* and *D. cristata*, and a single origin of *D. carthusiana*. Stein et al. [48], using chloroplast restriction site analyses, found no evidence to support multiple origins of either *D. cristata* or *D. carthusiana*. Werth also suggested a single origin for *D. celsa* based on isozyme analyses [82]. Surprisingly, we found no evidence from our sequence data to support multiple origins of any of the North American allopolyploids. For each species, the sequences of the A and B (and C, in the case of *D. clintoniana*) homeologs formed groups in which all accessions were each others’ closest relatives, and each group shared a single most recent common ancestor with one inferred diploid parental species, or group of species (Figure 3). No single accession had homeologs that were more similar to one individual of the inferred parental species, and this was the case for both nuclear markers employed here. The divergence time analysis (Figure 4) appears to depict separate origins of several of the allopolyploids, but this is a relict of the analysis, which will always produce a fully-resolved topology even among sequences where there are hard polytomies [94]. One exception is *D. campyloptera*; for this species, the B homeologs were identical in sequence to each other and to all sequences from the various *D. expansa* accessions, for both *pgiC* and *gapCp*. The sequences of the A homeologs of *pgiC* were identical to all of the *D. intermedia* accessions, but the A homeologs of *gapCp* from the two *D. campyloptera* accessions each shared a single nucleotide polymorphism with separate individuals of *D. intermedia*. This is extremely weak evidence for multiple origins, but could reflect independent formation of these two *D. campyloptera* lineages. We cannot strongly support recurrent origins for this species, but we cannot necessarily rule them out. For the other allopolyploid species our results also do not completely rule out multiple origins, particularly in the case of *D. carthusiana* and *D. cristata*, for which we are obviously lacking sequence data from one of the putative parental species. For both of these taxa, however, homeologs representing the second genome, donated by an extant taxon, are also monophyletic (with the exception of sequences from additional putative descendants of the same progenitors, e.g. *D. aquilinoides, D. muenchii, D. remota*), lending support to a hypothesized single origin (Figures 3, 4). For all of the North American allopolyploids, extensive additional sampling of these species and their progenitors will be essential before recurrent formation can either be confidently confirmed or ruled out. We included only two accessions of *D. clintoniana, D. campyloptera,* and *D. filix-mas*, three of *D. celsa*, and four of *D. carthusiana.* The likelihood of establishing multiple origins will be greater if sampling is increased, and if increased sampling fails to uncover evidence of multiple origins, it will also increase our confidence in rejecting recurrent formation of these species.

Based on our divergence time analysis (Figure 4), we can estimate the age of first formation of each polyploid species. We infer that the youngest of the splits between a hybrid allopolyploid’s homeologs and its closest relatives serves as an estimate of the maximum age of formation of each allopolyploid [95] (Table 4). In cases where these nodes are unsupported or poorly resolved in the best ML topology (Figure 3), we rely on the youngest well-supported node and these estimates may thus be older than the actual dates of formation. For *D. filix-mas*, the divergence time analysis included only those potential progenitors for which we had sequences of *gapCp*. Our results should therefore be considered inconclusive, but based on the dates of divergence of *D. filix-mas*’s two homeologs from their closest relatives in our analysis, we infer that it formed within the last 5.3 Ma (Table 4).

For *D. carthusiana*, *D. cristata,* and *D. clintoniana*, the shared maternal, *“D. semicristata”* lineage split from its closest relatives nearly 26 Ma, and that genome could have been donated to the allotetraploids at any subsequent time. *D. carthusiana* and *D. cristata* diverged from each other 12 Ma, but this is not necessarily the date at which they formed. The paternal lineage of *D. cristata* diverged from its progenitor (*D. ludoviciana*) within the last 13.7 Ma, and *D. cristata* could thus have formed any time since 13.7 Ma. The youngest well-supported node at which *D. carthusiana* diverges from its second parent, *D. intermedia*, is 11.5 Ma, and we estimate this to be the earliest date of its formation (Table 4). The earliest well-supported divergence of *D. clintoniana* from its paternal progenitor, *D. goldiana*, occurred 7.9 Ma, and the nodes at which it diverges from *D. cristata* date to 13.7 and 7.3 Ma. We thus infer that *D. clintoniana* formed within the last 7.3 Ma (Table 4). These dates are somewhat older than those found by Sessa et al. [16] based on divergence time analysis using a plastid dataset, which indicated that *D. carthusiana* and *D. cristata* descended from a Eurasian species (the *“D. semicristata”* lineage) that had diverged from its closest relative ca. 10 Ma, with the polyploids having formed subsequent to that. Following their formation via hybridization and polyploidization, Sessa et al. [16] inferred that these species arrived separately in North America via a long-distance dispersal event at least 0.4 Ma and a vicariance event at least 2.3 Ma, respectively. *D. carthusiana* and *D. cristata* are both widespread in North America (Figure 7A), as well as in Europe and parts of western Asia [12], and as a result it has often been suggested that *“D. semicristata”* must have been distributed in Eurasia [14]. However, the second parents of the allopolyploids (*D. intermedia* and *D. ludoviciana*) are endemic to North America, which would suggest that *“D. semicristata”* occurred in the Americas, in close enough proximity to *D. intermedia* and *D. ludoviciana* to enable two separate hybridization events to produce the allotetraploids. That *D. muenchii* and *D. remota* also appear be descendants of *“D. semicristata”* based on our analyses adds additional pieces to the biogeographic puzzle, but does not help to resolve it: *D. muenchii* is endemic to Mexico [62], and *D. remota* to Eurasia [76], supporting respectively an American and a Eurasian range for *“D. semicristata”*. The most parsimonious explanation would seem to be that *“D. semicristata”* inhabited a similar range to that of *D. carthusiana* and *D. cristata*, and was present in both the Americas and Eurasia, allowing it to form hybrids in both locations.

**Figure 8:**
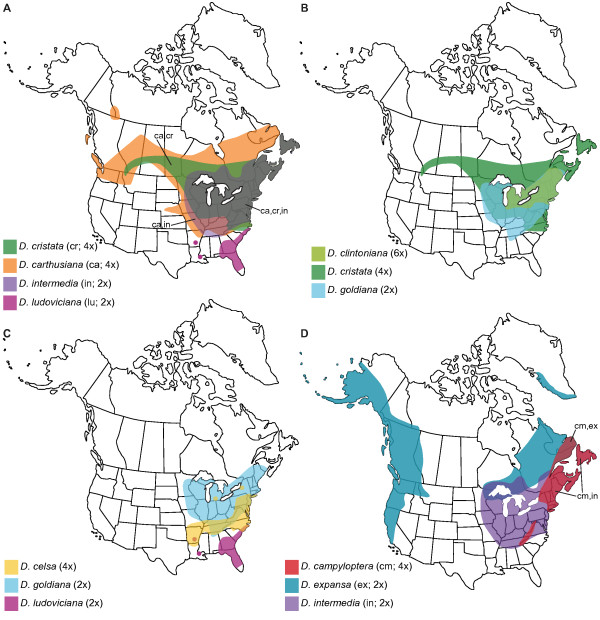
**Range maps for the North American allopolyploids and their putative parents.** Redrawn from [12]. Colors denote ranges of species, and areas of color overlap indicate that multiple species are present.

*D. campyloptera* diverged from its paternal parent, *D. expansa*, 4.6 Ma, and from its maternal parent, *D. intermedia*, 6.9 Ma, and thus we infer that it formed within the last 4.6 Ma (Table 4). For *D. campyloptera*, which is endemic to North America, our estimate of its earliest formation predates the estimated arrivals of its parental taxa in North America: 4.6 Ma compared to 0.2 and 0.9 Ma for *D. intermedia* and *D. expansa*, respectively [16]. However, the 95% HPD intervals on each of the relevant nodes overlap considerably, and the discrepancy in dates does not refute a North American origin for *D. campyloptera*. The final allotetraploid, *D. celsa,* diverged from its paternal parent, *D. goldiana,* 7.9 Ma and from its maternal parent, *D. ludoviciana*, 4.6 Ma, making its earliest possible date of formation 4.6 Ma (Table 4). One of *D. celsa*’s parental lineages had arrived on this continent by this time: Sessa et al. [16] estimated that *D. ludoviciana* had arrived in North American 5.6 Ma, while *D. goldiana* arrived ca 2.4 Ma. This suggests that *D. celsa*, which in endemic to North America, may have formed more recently than 2.4 Ma. Interestingly, the modern ranges of *D. celsa*’s progenitors do not overlap (Figure 8C), separated today by a ca. 240-km-wide corridor in the southeastern United States [12,14], and most ferns, including *Dryopteris*[96], have mobile spores that can readily disperse over distances similar to this. Within the last 2.4 Ma, the estimated period of *D. celsa*’s formation, the ranges of many plant species experienced considerable northward and southward shifts during periods of changing climate and glacial advance and retreat [97]. Such movements have been demonstrated for other plant groups (e.g. woody taxa, [98]), and would have provided extensive opportunities for intermixing of parental populations and formation of *D. celsa*.

Interestingly, the geographic ranges of the four North American allotetraploids are transgressive relative to the ranges of one or both of their parents (i.e. their ranges extend beyond those of their progenitors) (Figure 8A, C, D). This suggests that the allopolyploids may possess ecological or physiological advantages relative to their progenitors that have allowed them to colonize and persist in novel habitats or regions. In addition, the allotetraploids have all resulted from crosses between parents that display intermediate levels of genetic divergence compared to all potential pairs of progenitors (Figure 7). While a lower limit of inter-specific genetic divergence can be set at zero, there is no generally accepted upper value defining a high degree of divergence. However, species pairs in an intermediate or “goldilocks” zone of divergence would be expected to produce more successful allopolyploid offspring than pairs with either low or high levels of divergence, due to meiotic incompatibilities in the former and failures of fertilization in the latter [99,100]. Chapman and Burke [99] and Paun et al. [100] have reported correlations between genetic distance and polyploid incidence for numerous angiosperm genera, and Stelkens and Seehausen [101] have found divergence between parental species to be linked with transgressive trait expression across many hybrid eudicots. Such a relationship between genetic divergence and transgressive or advantageous physiological traits has thus far not been demonstrated in ferns, but may underlie the patterns we observe in *Dryopteris*. Ecological or physiological advantages driven by genetic divergence between parents may initially have allowed newly-formed allopolyploids to escape minority cytotype exclusion [92] following polyploidization, and over time may have led to stable, regional coexistence between the allopolyploid hybrids and their progenitors.

## Conclusions

The current study is the most comprehensive to date on the North American species of *Dryopteris*, which have long been suspected of having evolved via allopolyploid hybridization. Our analyses support all predictions of the “semicristata” hypothesis first proposed by Stanley Walker [13,22,23] for the parentage of the allopolyploids, and we reject several competing explanations for these species’ origins. Phylogenetic analyses of plastid sequence data allowed us to identify one parent of each of the allopolyploids, and our findings support a hypothesis for their parentage that includes the existence of a previously-proposed missing diploid progenitor taxon, *“D. semicristata”*[14]*.* Data from two nuclear markers confirm the identities of the second progenitors of each of the allopolyploids, and unambiguously support the “semicristata” hypothesis for their parentage. Copies of both markers from the descendants of *“D. semicristata”* grouped together in all analyses, and a reticulation network was unable to assign these sequences to an extant species’ genome.

The congruence between the two nuclear topologies presented in the current study confirms that hybridization rather than introgression accounts for the origins of the five allopolyploid species. We found no evidence for introgression between the allopolyploids and their progenitors, which is unsurprising given the difficulty of accomplishing gene flow across a ploidy barrier. However, deeper discordance between the topologies from different markers suggests that coalescent processes such as incomplete lineage sorting or ancient hybridization may have played a role in the evolution of the genus as a whole. Our sequence data failed to uncover evidence of multiple formations for any of the North American allopolyploids, which is surprising given that recurrent formation is now thought to be the norm in many polyploid lineages [86,87]. Our divergence time analyses established the earliest dates of formations of all of the North American allopolyploids as having occurred within the last ca. 14 Ma.

The current study demonstrates the utility of employing multiple genomic markers for addressing and untangling the evolutionary history of reticulate groups. This approach allowed us to identify maternal and paternal progenitors of all hybrid taxa, distinguish between allopolyploidization and introgression, test conflicting hypotheses for species’ origins, and confirm the existence of a “missing” diploid ancestor in a complex of plants that has long captivated and challenged systematics.

## Methods

### Taxon sampling & DNA extraction

All thirteen North American species of *Dryopteris* were included in this study, as were several species from other regions of the world that were found to be closely related to the North American group based on previous studies [16,17]. Multiple accessions of all species involved in the reticulate complex were included, for a total of 72 accessions representing 35 species (Table 2). For two species, *D. oreades* and *D. affinis*, we were unable to sequence *pgiC* from our single accession of each taxon, and so we included *pgiC* sequences obtained from Genbank, which were thus not derived from the same accession as the *gapCp* and plastid sequences reported here. We also included an additional *pgiC* sequence from *Dryopteris ludoviciana* that was obtained from Genbank, and which was the basis for a recent rejection of the “*D. semicristata*” hypothesis [49] (and see [17]). Two species of *Polystichum*, a genus closely related to *Dryopteris*[102,103], were included as outgroups. Tissue acquisition and DNA extraction procedures are described in [16].

### Plastid DNA sequencing

Plastid loci sequenced for this study included one protein-coding region (*rbcL*) and eight inter-genic spacers (*psbA-trnH, trnP-petG, rps4-trnS, trnL-F, trnG-trnR*, *rbcL-accD, trnV-trnM,* and *trnP-petG*). All regions except the last two were initially sequenced and reported in [16], and amplification and sequencing protocols are reported there. The same procedures were followed for *trnV-trnM* and *trnP-petG*, but the sequences are reported here for the first time. Primers used for polymerase chain reaction (PCR) and sequencing of all regions were based on previous studies (Table 3). Voucher information for all accessions is reported in Additional file Additional file 1: Table S1.

### Nuclear sequencing

*pgiC and gapCp* were initially amplified from all samples using PCR and existing primers [72,73]. For *pgiC*, primers 14F and 16R [72] are located in exons 14 and 16, resulting in amplification of portions of those exons, all of exon 15, and the intervening introns. *gapCp* primers 8F1 and 11R1 [73] are located in exons 8 and 11, and parts of those exons as well as all of exons 9 and 10 and the three intervening introns are amplified. Amplification occurred in 20 μL reactions containing 7.25 μL ddH_2_0, 4 μL 5x Colorless GoTaq Flexi buffer (Promega, Madison Wisconsin), 0.4 μL 10mM dNTP, 1 μL 25mM MgCl_2_, 2 μL of each 1mM primer, 0.25 μL GoTaq Flexi DNA polymerase (Promega, Madison, Wisconsin), and 3 μL template DNA diluted from stocks to 0.2 ng/μL. Amplifications were carried out on an Eppendorf MasterCycler Pro S (Eppendorf Scientific Inc., Hamburg, Germany) thermal cycler with the following protocols: for *pgiC,* 95°C for 7 min, (94°C for 30 s, 51°C for 1 min, 72°C for 1 min) × 40 cycles, 72°C for 4 min; for *gapCp,* 94°C for 3 min, (94°C for 1 min, 55°C for 1 min, 72°C for 3 min) × 35 cycles, 72°C for 10 min.

PCR products were run on a 1.2% agarose gel, from which bands were cut and DNA re-extracted using the ZymoClean Gel DNA Recovery System (Zymo Research Corp., Irvine, California). A single 700–800 base pair band was amplified and re-extracted in the *pgiC* reactions. There are two paralogs of *gapCp* in *Dryopteris*[73], a “long” copy and a “short” copy, which differ in the length of intron 9 and are easily separable on a gel. The “short” copy (600–900 bp) amplified reliably across most of our accessions and so was selected for these analyses.

We cloned both loci from all samples using the pGEM-T Easy Vector System I (Promega, Madison, Wisconsin) and following the protocol of [73] for cloning, colony selection, and post-cloning re-amplification with universal M13 primers. At least eight and up to 24 colonies were chosen for each individual. Final PCR products were purified using ExoSAP-IT (USB Corp., Cleveland, Ohio), and forward and reverse cycle-sequencing reactions carried out using BigDye Terminator 3.1 (Applied Biosystems, Foster City, California) with the region-specific primers. Sequencing products were purified via gel filtration chromatography using Sephadex columns (Sigma-Aldrich, St. Louis, Missouri) according to the manufacturer’s protocols. Sequencing occurred at the University of Wisconsin-Madison Biotechnology Center (Madison, Wisconsin).

Unique copies of *pgiC* and *gapCp* from all individuals were identified following [104] and [105]. Briefly, all sequences for a given accession were first pooled and observed by eye, and chimeric sequences easily identified and removed. An unrooted neighbor-joining tree was then constructed for each accession using the remaining sequences, and these trees were used along with visual inspection of the alignments to identify groups of sequences representing separate homeologs, which shared at least three polymorphisms (gaps, single, or multiple base pair changes). Consensus sequences were then constructed for these groups. We also retained singleton sequences that were not obviously chimeric or the result of PCR error, as they could potentially represent additional, under-sampled variation. Consensus and singleton sequences representing homeologs were assigned A, B, and C labels when more than one was found for a given species, and all sequences were deposited in GenBank (Appendix) and used in subsequent analyses.

### Sequence alignment and phylogenetic analyses

Alignment of the plastid sequences is described in [16]. *pgiC* and *gapCp* sequences were aligned using the MAFFT [106] plugin in Geneious 5.5.3 [107] and subsequently adjusted manually via the Geneious interface. Gaps in the alignments due to insertion/deletion events (indels) were coded as present or absent using the approach of [108] as implemented in the program FastGap [109], and appended to the nucleotide data as additional characters. Incongruence between the data partitions representing different portions of the plastid genome was assessed via the incongruence length difference (ILD) test [110], implemented as the partition homogeneity test in PAUP*4.0d102 [111]. When used correctly this method can be informative [112], though it is sensitive to a number of factors and can be prone to errors [113]. We therefore also visually compared trees reconstructed using individual partitions in order to identify any discordance between well supported clades.

Phylogenetic analyses were performed separately on the plastid*, pgiC,* and *gapCp* datasets using maximum parsimony (MP) in PAUPRat [114] and PAUP* [111], maximum likelihood (ML) in Garli 2.0 [115] and RAxML 7.2.8 [116,117], and Bayesian inference (BI) in MrBayes 3.1.2 [118]. PAUPRat, RAxML, and MrBayes analyses were conducted on the Cyberinfrastructure for Phylogenetic Research (CIPRES) Portal 2 ( http://www.phylo.org/portal2/) [55]. The amount of homoplasy in the data was evaluated using consistency indices, both including (CI) and excluding (CI’) autapomorphies [119].

MP analyses with PAUPRat, based on Parsimony Ratchet [120], were conducted using 1,000 ratchets with 200 iterations per replicate, following [121]. Support for clades was estimated using parsimony bootstrap analysis in PAUP* with 1,000 replicates, TBR branch swapping, simple taxon addition with one tree held at each step, and a maximum of 100 trees saved per replicate in order to decrease the time needed to run large bootstrap replicates. All MP analyses were run both with and without the indel data included, in order to assess their effects on topology and clade support. These data were not included in the ML and BI analyses, as CIPRES does not provide a way to model standard (non-nucleotide) variables in its analyses.

For ML and BI analyses, the optimal model of molecular evolution for each plastid and nuclear locus was identified using hierarchical likelihood ratio tests and the Akaike information criterion in MrModeltest 2.3 [122]. The most likely phylogeny for each dataset was produced in Garli 2.0 (Genetic Algorithm for Rapid Likelihood Inference) [115], using the optimal model of evolution for each partition. ML bootstrapping was executed in RAxML v. 7.2.8 (Randomized Accelerated Maximum Likelihood) [116,117]. The CIPRES portal allows only one model to be in place in RAxML analyses, though the dataset can be partitioned so that parameters for each partition may vary freely. Thus, for the plastid dataset, the most complex model for the set of loci was employed, and 1,000 bootstrap replicates were completed. BI analyses were completed in MrBayes 3.1.2 [118] on CIPRES, with different (optimal) models allowed for each region. Four independent runs of 10,000,000 generations were completed with four chains each (three heated, one cold), with a chain temp of 0.2 and uniform priors. Trees were sampled every 1,000 generations. Chain convergence and stationarity were assessed using Tracer 1.5 [123], by visually examining plots of parameter values and log-likelihood against the number of generations. Convergence and stationarity were assumed when the average standard deviation of split frequencies reached 0.01 or less. The first 25% of trees from each run were discarded as burn-in, and the remaining trees from the four runs were combined. A majority-rule consensus of these trees showing posterior probabilities (PP) was produced with PAUP*.

### Divergence time analysis

Divergence times were estimated for the *gapCp* dataset, for which we had the greatest number of accessions of North American species, using a Bayesian method [124] implemented in the program BEAST 1.6.2 (Bayesian Evolutionary Analysis by Sampling Trees; [125]). This method simultaneously estimates phylogeny and molecular rates using an MCMC strategy. The optimal GTR+Γ model of evolution was specified. We implemented a Yule process speciation prior and an uncorrelated lognormal (UCLN) model of rate change, with clock models unlinked between partitions. Analyses were run for 35,000,000 generations, with parameters sampled every 1,000 generations and the first 25% discarded as burnin. Tracer v1.5 [123] was used to examine the posterior distribution of all parameters and their associated statistics, including estimated sample sizes (ESS) and 95% highest posterior density (HPD) intervals. TreeAnnotator v1.5.4 [125] was used to summarize the set of post-burn-in trees and their parameters, in order to produce a maximum clade credibility (MCC) chronogram showing mean divergence time estimates with 95% HPD intervals. We implemented one calibration point, at the root node of *Dryopteris*, and modeled this as a lognormal prior with mean 2.0, stdev 0.5, and offset 35, in order to approximate the mean and 95% HPD intervals for the root of *Dryopteris* (42.4, 53.4-32.2 ma) found by Sessa et al. [16]. This secondary calibration point was employed due to a lack of reliable fossils of *Dryopteris* or Dryopteridaceae available for use in calibrating divergence time analyses (discussed in [16]). Lognormal priors, which apply a soft maximum bound with declining probability towards older dates [126], are particularly appropriate for use with secondary calibration points, as the distribution can account for some of the error associated with the original estimate [127,128].

### Reticulation network

A reticulation network showing inferred polyploidization events was constructed using the algorithm of [81] as implemented in the program PADRE [129,130]. The input data matrix consisted of sequences of *pgiC* and *gapCp* for one representative each of the thirteen North American *Dryopteris* species and all additional non-reticulate taxa present in our overall data set (i.e. all putatively diploid species that could potentially be progenitors of the North American allopolyploids) [95]. This matrix included 20 species of *Dryopteris* and one of *Polystichum*. We performed an ILD test to assess incongruence between the two nuclear markers for this set of taxa, and then obtained the best ML topology for the dataset using Garli 2.0 [115]. This multi-labelled topology was used as the input for PADRE.

### Genetic distances

For our plastid dataset, we calculated pairwise Jukes-Cantor distances between all known diploid, non-reticulate species present in our overall dataset. As in the reticulation network, this included all putatively diploid species that could potentially be progenitors of the North American allopolyploids. We inferred diploid sequences for *“D. semicristata”* by identifying the sequences from *D. carthusiana, D. cristata,* and *D. clintoniana* that we interpret as having been inherited from *“D. semicristata”* and taking their consensus. All pairwise genetic distances were ranked and a histogram of distances between all diploid pairs compiled, highlighting those five that corresponded to parental pairs that actually produced the North American allopolyploids (for *D. clintoniana*, the putative paternal progenitor is *D. goldiana*; the putative maternal parents is *D. cristata*, which is itself an allotetraploid whose putative maternal parent is *“D. semicristata”*, thus *D. goldiana–“D. semicristata”* was considered the parental pair for *D. clintoniana*). A randomization test with 10,000 replicates was conducted that assessed whether the sum of squared deviations from the mean (the overall mean of genetic distances for all pairs, equal to 0.0358) of the five parental pairs corresponding to the actual allopolyploids was significantly less than the random expectation. Significance (*P* < 0.05) was taken to be strong evidence for the “intermediate” nature of the genetic distances between parental pairs of the actual allopolyploids, in that in less than 5% of cases would a random set of five parental pair distances have a smaller sum of squared deviations from the mean than the set that gave rise to the five actual allopolyploids.

## Competing interests

The authors declare that they have no competing interests.

## Authors’ contributions

EBS obtained plant material from the field and herbaria, carried out all molecular work and subsequent analyses, and drafted the manuscript. TJG and EAZ participated in the design of the study, including taxon and analysis selection, and helped to draft the manuscript. All authors read and approved the final manuscript.

## Supplementary Material

Additional file 1: Table S1 Voucher information and GenBank accession numbers for all specimens included in this study. Click here for file
