# Supplementary material for: Unraveling reticulate evolution in North American Dryopteris (Dryopteridaceae)
Source: BMC Evol Biol. 2012 Jun 30;12:104. doi: 10.1186/1471-2148-12-104 (PMC3509404; doi:10.1186/1471-2148-12-104)
Supplement: Additional file 1: Table S1 — Voucher information and GenBank accession numbers for all specimens included in this study. [file 1471-2148-12-104-S1.pdf]

Additional file 1 – Voucher information and GenBank accession numbers for all specimens included in this study.
For species with more than one *pgiC* sequence, A, B, and/or C sequence labels are provided. Herbarium abbreviations: WIS = University of Wisconsin-Madison, Madison, Wisconsin, USA; NY = New York Botanical Garden, Bronx, New York, USA; DUKE = Duke University, Durham, North Carolina, USA; MO = Missouri Botanical Garden, St. Louis, Missouri, USA; UC = University of California-Berkeley, Berkeley, California, USA; COLO = University of Colorado Museum, Boulder, Colorado, USA. P = Muséum National d'Histoire Naturelle, Paris, France; BM = The Natural History Museum, London, UK; REU = Université de la Réunion, Saint-Clotilde, Réunion. AFSSE and BPSSE indicate taxa that were obtained as spores from the American Fern Society Spore Exchange and British Pteridological Society Spore Exchange, respectively. These spores were germinated and grown by Geiger and Ranker (2005), and DNA material later provided to us.

Taxon	Voucher (herbarium)	trnL-F	rbcL-accD	rbcL	psbA-trnH	rps4-trnS
***D. abbreviata* (C.Presl) Kuntze**	*Christenhusz 4290 (UC)*	JN189126	JN189664	JN189557	JN189448	JN189231
***D. affinis* (Lowe) Fraser Jenk.**	*Christenhusz 4281 (UC)*	JN189085	JN189626	JN189516	JN189408	JN189190
D. affinis* (Lowe) Fraser Jenk.	*Väre 16577*	—	—	—	—	—
***D. alpestris* Tagawa ex Ching & S.K.Wu**	*Heng 32147 (UC)*	JN189105	JN189645	JN189536	JN189428	JN189210
***D. antarctica* (Baker) C.Chr.**	*Hennequin 2009-R109 (REU)*	JN189141	JN189682	JN189577	JN189467	JN189250
***D. aquilinoides* C.Chr.**	*Kessler 13855 (UC)*	JN189106	JN189646	JN189537	JN189429	JN189211
***D. arguta* (Kaulf.) Maxon**	*EBS 35 (WIS)*	JN189077	JN189619	JN189509	JN189400	—
***D. arguta* (Kaulf.) Maxon**	*ESB 36 (WIS*	JN105310	JQ947924	JQ935258	JQ936649	JQ936838
***D. assimilis* S.Walker**	*Skvortsov 1.VIII.1982 (NY)*	JN189086	JN189627	JN189517	JN189409	JN189191
***D. austriaca* (Jacq.) Woyn. ex Schinz & Thell.**	*Degn 25 (NY)*	JN189087	JN189628	JN189518	JN189410	JN189192
***D. campyloptera* (Kuntze) Clarkson**	*EBS 19 (WIS)*	JN105306	JQ947921	JQ935255	JQ936639	JQ936819
***D. campyloptera* (Kuntze) Clarkson**	*EBS 22 (WIS)*	JN189072	JN189614	JN189504	JN189395	—
***D. carthusiana* (Vill.) H.P.Fuchs**	*EBS 7 (WIS)*	JQ682991	JQ947919	JQ935266	JQ936653	JQ936821
***D. carthusiana* (Vill.) H.P.Fuchs**	*EBS 41 (WIS)*	JN189079	JN189621	JN189511	JN189402	JN189184
***D. carthusiana* (Vill.) H.P.Fuchs**	*EBS 42 (WIS)*	JQ683006	JQ947923	JQ935254	JQ936661	JQ936839
***D. carthusiana* (Vill.) H.P.Fuchs**	*EBS 43 (WIS)*	JQ682990	JQ947938	JQ935272	JQ936647	JQ936843
***D. caucasica* (A.Braun) Fraser Jenk. & Corley**	*Christenhusz 4309 (UC)*	JN189109	JN189648	JN189540	JN189432	JN189214
***D. celsa* (W.Palmer) Knowlt., Palmer & Pollard**	*EBS 27 (WIS)*	JN189069	JN189609	JN189499	JN189390	JN189175
***D. celsa* (W.Palmer) Knowlt., Palmer & Pollard**	*EBS 49 (WIS)*	JN105314	JQ947917	JQ935249	JQ936641	JQ936822
***D. celsa* (W.Palmer) Knowlt., Palmer & Pollard**	*Price 94-2 (NY)*	JQ682981	—	—	JQ936652	JQ936840

Taxon	trnG-trnR	trnP-petG	matK	trnV	pgiC
D. abbreviata **(C.Presl) Kuntze**	JN189019	JN189019	—	JQ682942	JQ670060
D. affinis **(Lowe) Fraser Jenk.**	JN188980	JN188980	—	—	—
D. affinis **(Lowe) Fraser Jenk.***	—	—	—	—	FR728946.1 (A), FR728945.1 (B)
D. alpestris **Tagawa ex Ching & S.K.Wu**	JN189000	JN189000	JQ941627	JQ682961	JQ670044
D. antarctica **(Baker) C.Chr.**	JN189038	JN189038	JQ941648	JQ682971	JQ670026 (A), JQ670063 (B), JQ669996 (C)
D. aquilinoides **C.Chr.**	JN189001	JN189001	JQ941617	—	JQ670031 (A), JQ670002 (B)
D. arguta **(Kaulf.) Maxon**	JN188972	JN188972	JQ941647	JQ682958	JQ670099
D. arguta **(Kaulf.) Maxon**	JQ683022	JQ683022	JQ941660	JQ682977	JQ670052
D. assimilis **S.Walker**	JN188981	JN188981	JQ941622	JQ682966	JQ669971
D. austriaca **(Jacq.) Woyn. ex Schinz & Thell.**	JN188982	JN188982	JQ941637	JQ682972	JQ669993
D. campyloptera **(Kuntze) Clarkson**	JQ683013	JQ683013	JQ941619	JQ682921	JQ670015 (A), JQ669990 (B)
D. campyloptera **(Kuntze) Clarkson**	JN188967	JN188967	JQ941638	JQ682978	JQ670104 (A), JQ670100 (B)
D. carthusiana **(Vill.) H.P.Fuchs**	JQ683038	JQ683038	JQ941653	JQ682924	JQ670113 (A), JQ670096 (B)
D. carthusiana **(Vill.) H.P.Fuchs**	JN188974	JN188974	JQ941640	JQ682922	JQ670106 (A), JQ670108 (B)
D. carthusiana **(Vill.) H.P.Fuchs**	JQ683023	JQ683023	JQ941634	JQ682969	JQ670112 (A), JQ670116 (B)
D. carthusiana **(Vill.) H.P.Fuchs**	JQ683026	JQ683026	JQ941655	JQ682959	JQ669989 (A), JQ670050 (B)
D. caucasica **(A.Braun) Fraser Jenk. & Corley**	JN189004	JN189004	JQ941604	JQ682956	JQ670038 (A), JQ670018 (B)
D. celsa **(W.Palmer) Knowlt., Palmer & Pollard**	JN188962	JN188962	JQ941652	JQ682968	JQ670087 (A), JQ669967 (B)
D. celsa **(W.Palmer) Knowlt., Palmer & Pollard**	JQ683043	JQ683043	JQ941629	JQ682944	—
D. celsa **(W.Palmer) Knowlt., Palmer & Pollard**	JQ683035	JQ683035	JQ941658	JQ682917	—

Taxon	gapCp
D. abbreviata **(C.Presl) Kuntze**	—
D. affinis **(Lowe) Fraser Jenk.**	JQ936952 (A), JQ936885 (B)
D. affinis **(Lowe) Fraser Jenk.***	—
D. alpestris **Tagawa ex Ching & S.K.Wu**	JQ936924
D. antarctica **(Baker) C.Chr.**	JQ936925 (A), JQ936900 (B)
D. aquilinoides **C.Chr.**	JQ936919 (A), JQ936942 (B)
D. arguta **(Kaulf.) Maxon**	JQ936897
D. arguta **(Kaulf.) Maxon**	JQ936920
D. assimilis **S.Walker**	—
D. austriaca **(Jacq.) Woyn. ex Schinz & Thell.**	—
D. campyloptera **(Kuntze) Clarkson**	JQ936901 (A), JQ936962 (B)
D. campyloptera **(Kuntze) Clarkson**	JQ936961 (A), JQ936929 (B)
D. carthusiana **(Vill.) H.P.Fuchs**	JQ936964 (A), JQ936918 (B)
D. carthusiana **(Vill.) H.P.Fuchs**	JQ936915 (A), JQ936937 (B)
D. carthusiana **(Vill.) H.P.Fuchs**	—
D. carthusiana **(Vill.) H.P.Fuchs**	JQ936922 (A), JQ936928 (B)
D. caucasica **(A.Braun) Fraser Jenk. & Corley**	JQ936890 (A), JQ936889 (B)
D. celsa **(W.Palmer) Knowlt., Palmer & Pollard**	JQ936884
D. celsa **(W.Palmer) Knowlt., Palmer & Pollard**	JQ936949 (A), JQ936947 (B)
D. celsa **(W.Palmer) Knowlt., Palmer & Pollard**	JQ936955 (A), JQ936909 (B)

Taxon	Voucher (herbarium)	trnL-F	rbcL-accD	rbcL	psbA-trnH	rps4-trnS
D. chrysocoma **(Christ) C.Chr.**	*Unknown 188 (UC)*	JN189111	JN189650	JN189542	JN189434	JN189216
D. clintoniana **(D.C. Eaton) Dowell**	*EBS 16 (WIS)*	JN189068	JN189608	JN189498	JN189389	JN189174
D. clintoniana **(D.C. Eaton) Dowell**	*EBS 8 (WIS)*	JQ683004	JQ947934	JQ935247	JQ936651	JQ936813
D. crispifolia **Rasbach, Reichst. & G.Vida**	*BPSSE*	JN189164	JN189703	JN189597	JN189488	JN189272
D. cristata **(L.) A.Gray**	*ESB 26 (WIS)*	JQ682988	JQ947940	JQ935245	JQ936665	JQ936811
D. cristata **(L.) A.Gray**	*EBS 51 (WIS)*	JN189082	JN189623	JN189514	JN189405	JN189187
D. cristata **(L.) A.Gray**	*EBS 52 (WIS)*	JQ682995	—	JQ935267	JQ936669	JQ936834
D. cristata **(L.) A.Gray**	*Montgomery 07-99 (NY)*	JQ682987	—	JQ935251	—	JQ936835
D. cristata **(L.) A.Gray**	*Leoschke 2119 (NY)*	JQ682986	—	JQ935257	JQ936654	JQ936830
D. cristata **(L.) A.Gray**	*EBS 58 (WIS)*	JQ683002	JQ947928	JQ935263	JQ936662	JQ936832
D. cristata **(L.) A.Gray**	*EBS 68 (WIS)*	JQ682997	JQ947946	JQ935256	JQ936659	—
D. dilatata **(Hoffm.) A.Gray**	*Camoletto 2021 (NY)*	JQ682996	JQ947927	JQ935269	JQ936650	JQ936814
D. dilatata **(Hoffm.) A.Gray**	*Schuettpelz 535 (DUKE)*	JQ683001	JQ947918	JQ935276	JQ936648	JQ936841
D. dilatata **(Hoffm.) A.Gray**	*Hennequin 2010-B1 (P)*	JN189139	JN189680	JN189575	JN189465	JN189248
D. expansa **(C.Presl) Fraser Jenk. & Jermy**	*EBS 30 (WIS)*	JN189074	JN189616	JN189506	JN189397	JN189180
D. expansa **(C.Presl) Fraser Jenk. & Jermy**	*EBS 33 (WIS)*	JQ682999	JQ947937	JQ935270	JQ936672	JQ936816
D. expansa **(C.Presl) Fraser Jenk. & Jermy**	*EBS 37 (WIS)*	JQ682982	JQ947933	JQ935275	JQ936666	JQ936844
D. expansa **(C.Presl) Fraser Jenk. & Jermy**	*EBS 40 (WIS)*	JQ683000	JQ947935	JQ935250	JQ936645	JQ936820
D. filix-mas **(L.) Schott**	*EBS 32 (WIS)*	JN189075	JN189617	JN189507	JN189398	JN189181
D. filix-mas **(L.) Schott**	*EBS 38 (WIS)*	JQ683007	JQ947932	JQ935265	JQ936671	JQ936810
D. fragrans **(L.) Schott**	*EBS 47 (WIS)*	JN189080	—	JN189512	JN189403	JN189185
D. fragrans **(L.) Schott**	*EBS 53 (WIS)*	JQ682989	—	JQ935274	JQ936663	JQ936833
D. futura **A.R.Sm.**	*Quedensley 754 (UC)*	JN189103	JN189643	JN189534	JN189426	JN189208
D. goldiana **(Hook.) A.Gray**	*EBS 12 (WIS)*	JN105302	JQ947925	JQ935246	JQ936642	JQ936824
D. goldiana **(Hook.) A.Gray**	*EBS 24 (WIS)*	JQ683003	JQ947936	JQ935252	JQ936658	JQ936825
D. goldiana **(Hook.) A.Gray**	*EBS 29 (WIS)*	JN189073	JN189615	JN189505	JN189396	JN189179
D. goldiana **(Hook.) A.Gray**	*EBS 62 (WIS)*	JQ682984	JQ947926	—	JQ936644	JQ936836
D. goldiana **(Hook.) A.Gray**	*EBS 72 (WIS)*	JQ682993	JQ947941	JQ935260	JQ936664	JQ936818
D. guanchica **Gibby & Jermy**	*Hennequin 2010-C2 (P)*	JN189137	JN189678	JN189573	JN189463	JN189246
D. huberi **(Christ) C.Chr.**	*Sperling 5841 (NY)*	JN189089	JN189630	JN189520	JN189412	JN189194

Taxon	trnG-trnR	trnP-petG	matK	trnV	pgiC
D. chrysocoma **(Christ) C.Chr.**	JN189006	JN189006	JQ941620	JQ682927	JQ670083 (A), JQ669976, (B), JQ670028 (C)
D. clintoniana **(D.C. Eaton) Dowell**	JN188961	JN188961	JQ941605	JQ682934	—
D. clintoniana **(D.C. Eaton) Dowell**	JQ683019	JQ683019	JQ941626	JQ682920	JQ670066 (A), JQ669966 (B), JQ670064 (C)
D. crispifolia **Rasbach, Reichst. & G.Vida**	JN189057	JN189057	JQ941650	JQ682943	JQ670027 (A), JQ670057 (B)
D. cristata **(L.) A.Gray**	JQ683010	JQ683010	JQ941621	JQ682963	JQ670037 (A), JQ670025 (B)
D. cristata **(L.) A.Gray**	JN188977	JN188977	JQ941641	JQ682951	—
D. cristata **(L.) A.Gray**	JQ683011	JQ683011	JQ941661	JQ682935	—
D. cristata **(L.) A.Gray**	JQ683041	JQ683041	JQ941614	—	—
D. cristata **(L.) A.Gray**	JQ683012	JQ683012	JQ941609	JQ682919	—
D. cristata **(L.) A.Gray**	JQ683027	JQ683027	JQ941613	JQ682974	—
D. cristata **(L.) A.Gray**	JQ683020	JQ683020	JQ941608	JQ682947	—
D. dilatata **(Hoffm.) A.Gray**	JQ683025	JQ683025	JQ941645	JQ682954	—
D. dilatata **(Hoffm.) A.Gray**	JQ683034	JQ683034	JQ941599	JQ682931	JQ670092 (A), JQ670094 (B)
D. dilatata **(Hoffm.) A.Gray**	JN189036	JN189036	JQ941646	JQ682965	JQ669975 (A), JQ670020 (B)
D. expansa **(C.Presl) Fraser Jenk. & Jermy**	JN188969	JN188969	JQ941633	JQ682960	JQ669979
D. expansa **(C.Presl) Fraser Jenk. & Jermy**	JQ683030	JQ683030	JQ941610	JQ682946	JQ670110
D. expansa **(C.Presl) Fraser Jenk. & Jermy**	JQ683024	JQ683024	JQ941606	JQ682957	JQ670115
D. expansa **(C.Presl) Fraser Jenk. & Jermy**	JQ683018	JQ683018	JQ941612	JQ682955	JQ670103
D. filix-mas **(L.) Schott**	JN188970	JN188970	JQ941611	JQ682950	JQ670105
D. filix-mas **(L.) Schott**	JQ683036	JQ683036	JQ941618	JQ682939	JQ670041 (A), JQ670040 (B)
D. fragrans **(L.) Schott**	JN188975	JN188975	JQ941603	JQ682916	JQ670101
D. fragrans **(L.) Schott**	JQ683014	JQ683014	JQ941625	—	JQ669984
D. futura **A.R.Sm.**	JN188998	JN188998	—	JQ682962	JQ670070 (A), JQ670082 (B), JQ670022 (C)
D. goldiana **(Hook.) A.Gray**	JQ683040	JQ683040	JQ941601	JQ682973	—
D. goldiana **(Hook.) A.Gray**	JQ683021	JQ683021	JQ941600	JQ682930	JQ670091
D. goldiana **(Hook.) A.Gray**	JN188968	JN188968	JQ941644	JQ682928	JQ670073
D. goldiana **(Hook.) A.Gray**	JQ683029	JQ683029	JQ941615	JQ682970	—
D. goldiana **(Hook.) A.Gray**	JQ683015	JQ683015	JQ941602	JQ682949	JQ670111
D. guanchica **Gibby & Jermy**	JN189034	JN189034	—	JQ682938	JQ669959 (A), JQ670076 (B)
D. huberi **(Christ) C.Chr.**	JN188984	JN188984	—	—	JQ670006 (A), JQ669995 (B), JQ670001 (C)

Taxon	gapCp
D. chrysocoma **(Christ) C.Chr.**	JQ936895 (A), JQ936898 (B)
D. clintoniana **(D.C. Eaton) Dowell**	JQ936921 (A), JQ936935 (B), JQ936965 (C)
D. clintoniana **(D.C. Eaton) Dowell**	JQ936923 (A), JQ936910 (B), JQ936912 (C)
D. crispifolia **Rasbach, Reichst. & G.Vida**	—
D. cristata **(L.) A.Gray**	JQ936886 (A), JQ936945 (B)
D. cristata **(L.) A.Gray**	JQ936943 (A), JQ936916 (B)
D. cristata **(L.) A.Gray**	JQ936963 (A), JQ936899 (B)
D. cristata **(L.) A.Gray**	JQ936891 (A), JQ936956 (B)
D. cristata **(L.) A.Gray**	JQ936930 (A), JQ936926 (B)
D. cristata **(L.) A.Gray**	JQ936951
D. cristata **(L.) A.Gray**	JQ936896
D. dilatata **(Hoffm.) A.Gray**	JQ936902
D. dilatata **(Hoffm.) A.Gray**	JQ936917 (A), JQ936892 (B)
D. dilatata **(Hoffm.) A.Gray**	—
D. expansa **(C.Presl) Fraser Jenk. & Jermy**	JQ936908
D. expansa **(C.Presl) Fraser Jenk. & Jermy**	JQ936907
D. expansa **(C.Presl) Fraser Jenk. & Jermy**	JQ936954
D. expansa **(C.Presl) Fraser Jenk. & Jermy**	—
D. filix-mas **(L.) Schott**	JQ936957 (A), JQ936946 (B)
D. filix-mas **(L.) Schott**	JQ936953 (A), JQ936914 (B)
D. fragrans **(L.) Schott**	JQ936905
D. fragrans **(L.) Schott**	JQ936893
D. futura **A.R.Sm.**	—
D. goldiana **(Hook.) A.Gray**	JQ936936
D. goldiana **(Hook.) A.Gray**	JQ936906
D. goldiana **(Hook.) A.Gray**	JQ936939
D. goldiana **(Hook.) A.Gray**	JQ936888
D. goldiana **(Hook.) A.Gray**	JQ936938
D. guanchica **Gibby & Jermy**	—
D. huberi **(Christ) C.Chr.**	—

Taxon	Voucher (herbarium)	trnL-F	rbcL-accD	rbcL	psbA-trnH	rps4-trnS
D. intermedia **Kuntze**	*ESB 15 (WIS)*	JQ683008	JQ947930	JQ935268	JQ936668	JQ936829
D. intermedia **Kuntze**	*EBS 13 (WIS)*	JN105304	JQ947944	JQ935261	JQ936640	JQ936842
D. intermedia **Kuntze**	*EBS 18 (WIS)*	—	JN189613	JN189503	JN189394	JN189178
D. intermedia **Kuntze**	*EBS 44 (WIS)*	JQ682985	JQ947942	JQ935248	JQ936657	JQ936831
D. intermedia **Kuntze**	*EBS 63 (WIS)*	JQ682980	JQ947945	JQ935271	JQ936670	JQ936817
D. intermedia **Kuntze**	*EBS 69 (WIS)*	JQ682992	JQ947931	JQ935262	JQ936643	JQ936812
D. ludoviciana **(Kunze) Small**	*EBS 48 (WIS)*	JN189081	JN189622	JN189513	JN189404	JN189186
D. ludoviciana **(Kunze) Small**	*EBSlud2 (WIS)*	JQ682979	JQ947939	JQ935264	JQ936667	JQ936828
D. ludoviciana **(Kunze) Small**	*EBSlud3 (WIS)*	JN105313	JQ947943	JQ935277	JQ936656	JQ936815
D. ludoviciana **(Kunze) Small**	*EBSlud4 (WIS)*	JQ683005	JQ947929	JQ935259	JQ936660	JQ936823
D. ludoviciana **(Kunze) Small**	*EBS 48 (WIS)*	JQ682994	JQ947920	JQ935273	JQ936673	JQ936837
D. ludoviciana **(Kunze) Small ***	*Leonard 2144*	—	—	—	—	—
D. marginalis **(L.) A.Gray**	*EBS 17 (WIS)*	JN189071	JN189612	JN189502	JN189393	JN189177
D. monticola **(Makino) C.Chr.**	*Togasi (COLO)*	JN189156	—	—	JN189482	JN189264
D. muenchii **A.R.Sm.**	*Hoshizaki (UC)*	JQ682998	—	—	—	JQ936827
D. muenchii **A.R.Sm.**	*EBS 54 (WIS)*	JN189104	JN189644	JN189535	JN189427	JN189209
D. oligodonta **Pic. Serm.**	*Hennequin 2010-C11 (P)*	JN189138	JN189679	JN189574	JN189464	JN189247
D. oreades **Fomin**	*Vasak 427039 (COLO)*	JN189146	—	—	JN189472	JN189254
D. oreades **Fomin ***	*Kukkonon 13904*	—	—	—	—	—
D. pallida **Fomin**	*AFSSE*	JN189158	JN189697	JN189591	—	JN189266
D. remota **Hayata**	*Schuettpelz 528 (DUKE)*	JN189099	JN189640	JN189530	JN189422	JN189204
D. remota **Hayata**	*Moran (COLO)*	JQ682983	JQ947922	JQ935253	JQ936655	JQ936826
D. scottii **(Bed) Ching**	*RBC 202 (UC)*	JN189121	JN189659	JN189552	JN189444	JN189226
D. tokyoensis **(Matsum.) C.Chr.**	*Moran (COLO)*	JN189142	JN189683	JN189578	JN189468	JN189251
Polystichum andersonii **Hopkins**	*EBS 39 (WIS)*	JN189078	JN189620	JN189510	JN189401	JN189183
Polystichum munitum **(Kaulf.) C. Presl**	*EBS 34 (WIS)*	JN189076	JN189618	JN189508	JN189399	JN189182

* *pgiC* sequence obtained from GenBank.

Taxon	trnG-trnR	trnP-petG	matK	trnV	pgiC
D. intermedia **Kuntze**	JQ683017	JQ683017	JQ941624	JQ682952	JQ670095
D. intermedia **Kuntze**	JQ683039	JQ683039	JQ941663	JQ682925	JQ670097
D. intermedia **Kuntze**	JN188966	JN188966	JQ941630	JQ682918	JQ670071
D. intermedia **Kuntze**	JQ683009	JQ683009	JQ941659	JQ682975	JQ670093
D. intermedia **Kuntze**	JQ683032	JQ683032	JQ941657	JQ682936	—
D. intermedia **Kuntze**	JQ683037	JQ683037	JQ941642	JQ682941	JQ670109
D. ludoviciana **(Kunze) Small**	JN188976	JN188976	JQ941654	JQ682945	—
D. ludoviciana **(Kunze) Small**	JQ683031	JQ683031	JQ941656	JQ682953	JQ670107
D. ludoviciana **(Kunze) Small**	JQ683028	JQ683028	JQ941628	JQ682964	JQ670014
D. ludoviciana **(Kunze) Small**	JQ683016	JQ683016	JQ941639	JQ682937	JQ670090
D. ludoviciana **(Kunze) Small**	JQ683033	JQ683033	JQ941631	JQ682940	JQ670114
D. ludoviciana **(Kunze) Small ***	—	—	—	—	FR728995.1
D. marginalis **(L.) A.Gray**	JN188965	JN188965	JQ941607	JQ682932	JQ670012
D. monticola **(Makino) C.Chr.**	—	—	—	—	JQ670019 (A), JQ670036 (B)
D. muenchii **A.R.Sm.**	—	—	—	—	JQ670089 (A), JQ670102 (B), JQ670098 (C)
D. muenchii **A.R.Sm.**	JN188999	JN188999	JQ941632	—	JQ670042 (A), JQ670007 (B), JQ669992 (C)
D. oligodonta **Pic. Serm.**	JN189035	JN189035	JQ941643	JQ682926	JQ669960
D. oreades **Fomin**	—	—	—	—	—
D. oreades **Fomin ***	—	—	—	—	FR729003.1
D. pallida **Fomin**	—	—	JQ941649	JQ682976	JQ670081
D. remota **Hayata**	JN188994	JN188994	JQ941635	JQ682933	—
D. remota **Hayata**	JQ683042	JQ683042	JQ941616	JQ682948	JQ670080 (A), JQ670056 (B)
D. scottii **(Bed) Ching**	JN189015	JN189015	JQ941623	JQ682967	JQ669964 (A), JQ670077 (B)
D. tokyoensis **(Matsum.) C.Chr.**	JN189039	JN189039	JQ941651	—	JQ670021
Polystichum andersonii **Hopkins**	JN188973	JN188973	JQ941662	JQ682929	JQ669978
Polystichum munitum **(Kaulf.) C. Presl**	JN188971	JN188971	JQ941636	JQ682923	—

Taxon	gapCp
D. intermedia **Kuntze**	JQ936933
D. intermedia **Kuntze**	—
D. intermedia **Kuntze**	JQ936904
D. intermedia **Kuntze**	JQ936934
D. intermedia **Kuntze**	JQ936948
D. intermedia **Kuntze**	JQ936950
D. ludoviciana **(Kunze) Small**	JQ936940
D. ludoviciana **(Kunze) Small**	—
D. ludoviciana **(Kunze) Small**	JQ936927
D. ludoviciana **(Kunze) Small**	—
D. ludoviciana **(Kunze) Small**	JQ936958
D. ludoviciana **(Kunze) Small ***	—
D. marginalis **(L.) A.Gray**	JQ936894
D. monticola **(Makino) C.Chr.**	—
D. muenchii **A.R.Sm.**	JQ936960 (A), JQ936959 (B), JQ936931 (C)
D. muenchii **A.R.Sm.**	JQ936932 (A), JQ936911 (B), JQ936903 (C)
D. oligodonta **Pic. Serm.**	—
D. oreades **Fomin**	—
D. oreades **Fomin ***	—
D. pallida **Fomin**	—
D. remota **Hayata**	JQ936941 (A), JQ936944 (B)
D. remota **Hayata**	—
D. scottii **(Bed) Ching**	—
D. tokyoensis **(Matsum.) C.Chr.**	JQ936913
Polystichum andersonii **Hopkins**	—
Polystichum munitum **(Kaulf.) C. Presl**	JQ936887
